# Contemporary Techniques and Prospects in Pharmaceutical Tablet Surface Analysis

**DOI:** 10.1021/acsmeasuresciau.5c00178

**Published:** 2026-02-02

**Authors:** Matjaž Finšgar

**Affiliations:** Faculty of Chemistry and Chemical Engineering, University of Maribor, 2000 Maribor, Slovenia

**Keywords:** ToF-SIMS, XPS, AFM, 3D-profilometry, pharmaceutical tablet, solid dosage forms

## Abstract

This work demonstrates how surface analysis can be applied for the chemical characterization of solid pharmaceutical tablets using time-of-flight secondary ion mass spectrometry (ToF-SIMS), X-ray photoelectron spectroscopy (XPS), atomic force microscopy (AFM), and 3D profilometry as complementary tools. Two formulation extremes were examined, i.e., high-dose single active pharmaceutical ingredient (API) tablets and low-dose three-API combination tablets, with five formulations assessed in each set. AFM and 3D profilometry were employed to characterize the micro- to nanoscale topography, assess roughness, and measure the crater depths formed after gas cluster ion beam (GCIB) sputtering. To define ToF-SIMS markers, API reference standards were analyzed using tandem (MS/MS) ToF-SIMS. Multivariate curve resolution was used to identify ions unique to each API. After marker definition, ToF-SIMS images were acquired in 2D and, by using GCIB, in 3D. Large-area maps were produced by image stitching. Delayed extraction and fast imaging modes enabled submicrometric imaging at high mass resolving power. XPS survey and high-resolution spectra, combined with GCIB sputtering, quantified the elemental composition and chemical states within the outer few nanometers and into the subsurface region. It was demonstrated that ToF-SIMS can provide molecularly specific maps and depth profiles that localize APIs and excipients, revealing surface segregation and interfacial layering. In contrast, XPS supplies quantitative elemental and chemical-state information on the surface and in the subsurface. Overall, the study demonstrates that these surface analytical techniques offer spatially resolved insights not accessible with conventional methods for solid dosage forms and that they complement practices in formulation development, troubleshooting, and quality control. These techniques can confirm API and excipient localization, assess surface segregation and interfacial layers, detect contaminants, and compare batches. Despite this utility, they have seen limited adoption, most likely because they require specialized instrumentation, method development, and data interpretation expertise not yet widespread in pharmaceutical laboratories.

## Graphical abstract

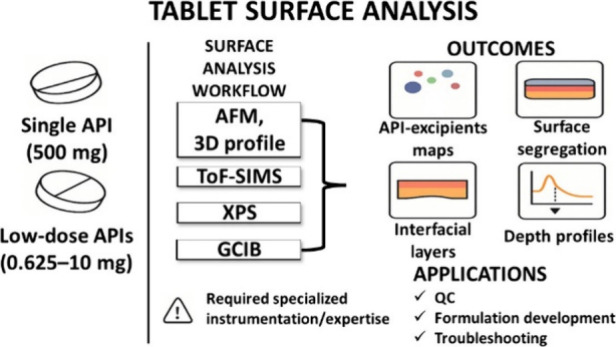

## 1 Introduction

Tablets are a widely used dosage form, but their critical quality attributes, such as active pharmaceutical ingredient (API) content, distribution, hardness, and dissolution, are typically assessed by time-consuming and labor-intensive methods that often lack spatial information.^1^ Current solid-dose tablet analysis increasingly combines vibrational spectroscopies with imaging to quantify composition and map the distribution of APIs. In practice, Raman and Fourier transform infrared (FTIR) spectroscopies are the primary techniques used. Both provide rich, molecularly selective or molecularly specific spectra, and both can discriminate between crystal forms (polymorphs) when the full spectrum is considered rather than a single peak, typically via multivariate statistical analysis (MVSA) methods to handle overlap and matrix effects.^2^ For the spatial mapping of APIs and excipients, Raman spectroscopy chemical imaging offers (sub)­micron-scale lateral detail and is routinely used by acquiring a spectrum at each pixel and comparing such against spectral libraries. FTIR spectroscopy imaging achieves lower spatial detail (several to tens of micrometers) but remains valuable for chemically specific mapping over larger areas.^2b^ Confocal Raman spectroscopy can provide depth-resolved 3D maps, but is fundamentally limited by optical transparency and scattering, which restricts the practical depths in turbid tablets.^3^ Moreover, coupling Raman and FTIR spectroscopies to the topography of the sample improves interpretation. Thus, atomic force microscopy (AFM) coupled with Raman spectroscopy enables the correlation of nanoscale topography with chemical identity, thereby mitigating topography-induced spectral artifacts during mapping. Furthermore, X-ray-based techniques can complement Raman and FTIR spectroscopies in such studies. Scanning electron microscopy with energy-dispersive X-ray spectroscopy (SEM-EDXS) can generate element-specific maps, which can be useful for locating certain excipients (for example, the distribution of Mg from Mg stearate^4^). However, the use of such a technique is limited because elements like Mg may originate from multiple sources. For instance, both Mg stearate and talc are common excipients, making it impossible to assign the signal unambiguously. Other X-ray-based techniques, such as microcomputed tomography, provide complementary insight by probing internal structure and porosity but do not yield detailed chemical information.^5^

Time-of-flight secondary ion mass spectrometry (ToF-SIMS) and X-ray photoelectron spectroscopy (XPS) offer complementary surface analysis capabilities for tablet characterization, which remain largely underutilized in routine practice. ToF-SIMS, although still rarely applied to map APIs in tablets, probes only the upper few nanometers of the surface. It can localize molecular fragments and molecular ions with a lateral resolution of approximately 100 nm (a routinely achievable lateral resolution in organic matrices). In applying ToF-SIMS, one must balance mass resolution, lateral resolution, and acquisition time according to the analytical objective. XPS also probes the outer few nanometers and yields (semi)­quantitative elemental composition and short-range chemical-state information. Complementary to the latter, ToF-SIMS contributes to molecular specificity and molecular selective imaging. Both techniques can access subsurface regions using gas cluster ion beam (GCIB) sputtering, enabling the chemically nondestructive depth profiling of organic components.^6^ The results obtained from GCIB sputtering, combined with ToF-SIMS and XPS, can help assess surface segregation, interfacial layers, and the heterogeneity of APIs and excipients. Additionally, they can detect the presence of trace contaminants and support development, reverse engineering, and quality control. AFM provides topographical and nanomechanical context, including roughness, phase contrast, and local heterogeneity, which enhances region selection and the interpretation of XPS and ToF-SIMS data.

Paracetamol (PAR) is a widely used analgesic and antipyretic. In contrast, indapamide (IND), amlodipine (AMLO), and perindopril (PER) are antihypertensive drugs classified as a thiazide-like diuretic, a dihydropyridine calcium channel blocker, and an angiotensin-converting enzyme inhibitor, respectively.

In order to illustrate the challenges of employing surface analysis techniques for the characterization of analytically complex samples, two extremes of API loading were examined: (i) single-API PAR tablets containing 500 mg PAR, representative of high-dose formulations; and (ii) combination (COMB) tablets containing a low dose of IND, AMLO, and PER in the 0.625–10 mg range, representative of low-level APIs dispersed within a larger excipient matrix. This design enables the evaluation of how surface analysis techniques respond to high and low API content, as well as the assessment of API distribution and excipient interactions under both conditions.

The main objective of this work is to present the current state of applying ToF-SIMS and XPS in the analysis of pharmaceutical tablets. In addition, AFM and 3D profilometry are employed to provide complementary insights into the tablet surface topography and roughness at both micro- and nanoscale levels. Surface analysis methods are extremely rarely used in pharmaceutical tablet analysis. Yet, this study demonstrates how they can be employed to probe both the surface and subsurface regions, reveal formulation-dependent differences, and capture information inaccessible by conventional techniques, e.g., Raman spectroscopy. Moreover, the limitations of these approaches are addressed, highlighting the practical considerations needed for their broader application in pharmaceutical research and quality control.

## 2 Experimental Section

### 2.1 3D Profilometry and AFM Measurements

Macroscopic surface topography and crater depths generated after GCIB sputtering in XPS and ToF-SIMS experiments were characterized using a DektakXT stylus profilometer (Bruker, Karlsruhe, Germany). The acquired 3D profiles were postprocessed with MountainsMap Imaging Topography software (Digital Surf, Besançon, France).

For nanoscale surface characterization, AFM analyses were carried out on an MFP-3D Origin Plus instrument (Asylum Research/Oxford Instruments, Santa Barbara, CA, USA). Measurements were performed in tapping mode using OMCL AC240TS-C3 aluminum-coated probes (Olympus), with a resonant frequency of 70 kHz and a nominal spring constant of 1.7 N/m. Scan rates were typically 0.65–0.75 Hz, with an image resolution of 256 × 256 pixels. Image rendering was completed using Igor Pro software.

### 2.2 ToF-SIMS Measurements

All ToF-SIMS measurements were performed on a ToF-SIMS instrument supplied by IONTOF GmbH (Münster, Germany, Model M6) equipped with a Bi liquid metal ion gun (LMIG, Nanoprobe 50) and a GCIB source. The primary ion source was a pulsed Bi_3_^+^ beam directed at the sample at a 45° angle (30 keV, with a target current of 0.6 pA), which was used for both spectral acquisition and imaging. For depth profiling, unless stated otherwise, a 10 keV Ar_2000_^+^ GCIB (with a target current of 10 nA) was employed. The use of GCIB sputtering enables the gradual removal of surface layers, while preserving the chemical information on the organic species, making it suitable for distinguishing active ingredients and excipients in complex pharmaceutical formulations. The GCIB sputter beam position was checked and calibrated prior to performing depth profile measurements.

Charge compensation was applied using a 20 eV electron flood gun and 5·10^–7^ mbar Ar gas flooding. Surface potential was applied as required. The MS^2^ ToF-SIMS spectra were acquired with the collision cell operated at 1 · 10^–6^ mbar (He) and a collision energy of 2000 V.

For all PAR tablets, mass calibration was performed using the signals at a mass-to-charge ratio (*m*/*z*) of 27.02 for C_2_H_3_^+^, *m*/*z* 29.04 for C_2_H_5_^+^, *m*/*z* 41.04 for C_3_H_5_^+^, *m*/*z* 43.05 for C_3_H_7_^+^, *m*/*z* 53.04 for C_4_H_5_^+^, *m*/*z* 55.05 for C_4_H_7_^+^, *m*/*z* 57.07 for C_4_H_9_^+^, and *m*/*z* 69.07 for C_5_H_9_^+^. For all COMB tablets, the mass calibration was performed using signals at an *m*/*z* of 29.04 for C_2_H_5_^+^, *m*/*z* 41.04 for C_3_H_5_^+^, *m*/*z* 43.05 for C_3_H_7_^+^, *m*/*z* 53.04 for C_4_H_5_^+^, *m*/*z* 55.05 for C_4_H_7_^+^, *m*/*z* 57.07 for C_4_H_9_^+^, and *m*/*z* 69.07 for C_5_H_9_^+^. Mass calibration in delayed extraction analyzer mode was performed with signals above *m*/*z* 40.00.

Intact tablets, taken directly from the original foil packaging and handled to avoid contact with the analyzed surface, were examined without cutting or further preparation and mounted on a backmount sample holder. Spectra, imaging, and data evaluation were performed using SurfaceLab 7.5 (IONTOF GmbH).

### 2.3 XPS Measurements

XPS analyses were carried out on a Kratos Supra+ spectrometer (Kratos Analytical, Manchester, UK) equipped with a monochromatic Al K_α_ radiation source. All spectra were referenced to the C–C/C–H contribution in the C 1s spectra at 284.8 eV. Surface charging was compensated for by a low-energy electron flood gun. Data collection and processing were performed using the ESCApe 1.5 software package (Kratos). Quantification was performed after Shirley background subtraction.

Tablets were mounted on the sample holder using double-sided, Si-free adhesive tape. Measurements were performed at a take-off angle of 90° with a spot size of 300 μm by 700 μm. Survey spectra were acquired with a pass energy of 160 eV, while high-resolution (HR) spectra were collected with a pass energy of 40 eV. Depth profiling was performed by GCIB sputtering with 10 keV Ar_1000_^+^ over a 3.0 mm by 3.0 mm area. The GCIB sputter beam position was checked and calibrated prior to performing depth profile measurements.

### 2.4 Analytes

Table 1 summarizes the tablets analyzed in this study. The PAR tablets were produced by five different manufacturers, originating from various countries. Each contained 500 mg of PAR as the API, but the excipient composition differed between formulations. The COMB tablets, produced by a single manufacturer, contained varying amounts of IND, AMLO, PER, and the same excipient make up. COMB denotes tablets containing a combination of different APIs in a single formulation. For all PAR and COMB tablet formulations, the manufacturers did not disclose the quantities of excipients.

**Table 1:** Summary of the PAR and COMB Tablet Formulations as Declared by Manufacturers and Analyzed in This Study

**Designation**	**API**	**Excipients**
**PAR tablets**		
PAR 1	500 mg PAR	methyl p-hydroxybenzoate, propyl p-hydroxybenzoate, gelatin, SiO_2_, talc, Mg stearate
PAR 2	500 mg PAR	povidone, sodium starch glycolate, stearic acid
PAR 3	500 mg PAR	croscarmellose sodium, maize starch, cellulose, povidone, talc, SiO_2_, Mg stearate
PAR 4	500 mg PAR	pregelatinized starch, povidone, stearic acid
PAR 5	500 mg PAR	maize starch, povidone, talc, SiO_2_, Mg stearate, formaldehyde caseinate, potassium sorbate
**COMB tablets**		
COMB 1	2 mg PER, 5 mg AMLO, 0.625 mg IND	sodium hydrogen carbonate, cellulose, maize starch, sodium carboxymethyl starch, SiO_2_, Mg stearate, and CaCl_2_
COMB 2	4 mg PER, 5 mg AMLO, 1.25 mg IND	same excipient composition as COMB 1
COMB 3	4 mg PER, 10 mg AMLO, 1.25 mg IND	same excipient composition as COMB 1
COMB 4	8 mg PER, 5 mg AMLO, 2.5 mg IND	same excipient composition as COMB 1
COMB 5	8 mg PER, 10 mg AMLO, 2.5 mg IND	same excipient composition as COMB 1

Reference standards, featured as white powders, were purchased as certified reference material from Sigma-Aldrich (St. Louis, MO, USA); PAR (CAS-No: 103–90–2), IND (CAS-No: 26807–65–8), AMLO (CAS-No: 111470–99–6), and PER (as perindopril erbumine, CAS-No: 107133–36–8). The reference standard powders were compacted into tablets using a Specac Atlas 25T hydraulic press (Orpington, UK), fitted with 13 mm stainless steel discs, with a force of 5 tons applied at room temperature. This produced mechanically stable tablets suitable for ToF-SIMS and XPS measurements, without the addition of any binders or auxiliary materials.

## 3 Results and Discussion

### 3.1 Surface Analysis of Pharmaceutical Tablets Using 3D Profilometry and AFM

3D profilometry provides microscale information on tablet surface topography by scanning with a stylus to generate height maps. This enables the assessment of the curvature, uniformity, and mechanical defects relevant to the dissolution behavior of the tablets. From these maps, roughness parameters, such as mean surface roughness (*S*_a_), can be extracted. A higher *S*_a_ indicates a rougher surface with more pronounced roughness, which can enhance the wetting and dissolution of the tablets but may also promote any mechanical weakness or variability in coating and lubrication. A lower *S*_a_ reflects a smoother, more uniform surface, typically associated with improved mechanical integrity and reproducibility, but potentially slower initial wetting or dissolution of the tablets. The acquired 3D profiles can be further processed using so-called form removal, a software function that corrects for the intrinsic convex or concave tablet shape, allowing for a truer evaluation of microroughness and sputter depth (this operation was applied to the profiles in Figure 1, S1, and S2a). 3D profilometry also yields crater geometry, particularly depth after sputtering, which is essential for calibrating depth scales in GCIB-XPS and GCIB-ToF-SIMS (an example of such a crater formed after ToF-SIMS sputtering is given in Figure S1).

**Figure 1:**
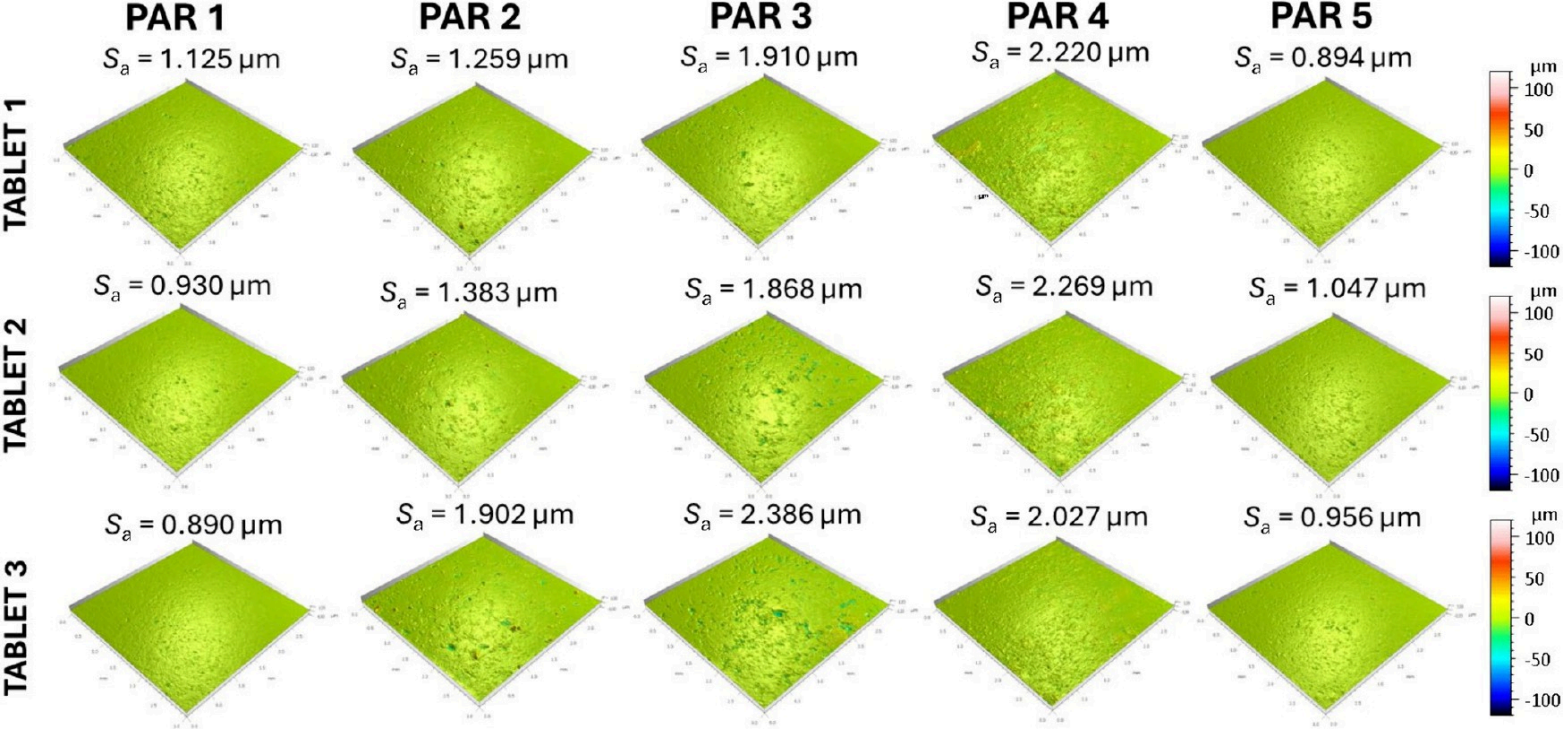
3D profilometry measurements performed on three individual tablets from the same PAR manufacturer, with *S*_a_ values indicated. Measurements were performed on 3.0 mm by 3.0 mm spot sizes.

AFM provides nanoscale surface information that complements 3D profilometry and spectroscopy, yet its application in pharmaceutical tablet analysis remains limited. Height images reveal nanometer-scale roughness and microstructural features relevant to dissolution and mechanical stability (e.g., microcrystal formation or lubricant layers).^7^ Amplitude data enhances edge definition and permits the determination of segregated domains within heterogeneous excipient-API systems.^8^ Phase imaging provides insight into local variations in stiffness, adhesion, and viscoelasticity, thereby distinguishing between crystalline and amorphous regions or identifying domains enriched in lubricants, such as Mg stearate.^7,9^ Beyond imaging, AFM-based adhesion force measurements and force–distance spectroscopy can provide quantitative insights into excipient-API interactions, adhesive behavior of tablet tooling, and the effect of humidity on interfacial forces.^10^ These capabilities enable AFM to probe critical factors in formulation tablet science, including miscibility, phase distribution, and mechanical performance at the nanoscale [9–19], providing analytical information not attainable by other surface analytical techniques.

Examples of such analyses using 3D profilometry and AFM imaging are presented in Figures 1 and 2, as well as in Figures S2–S8. These results highlight differences both between formulations and within individual tablets, showing that the surfaces of the same tablet are not homogeneous. 3D profilometry highlights differences in large-scale roughness, while AFM provides nanoscale insight into surface organization.

**Figure 2:**
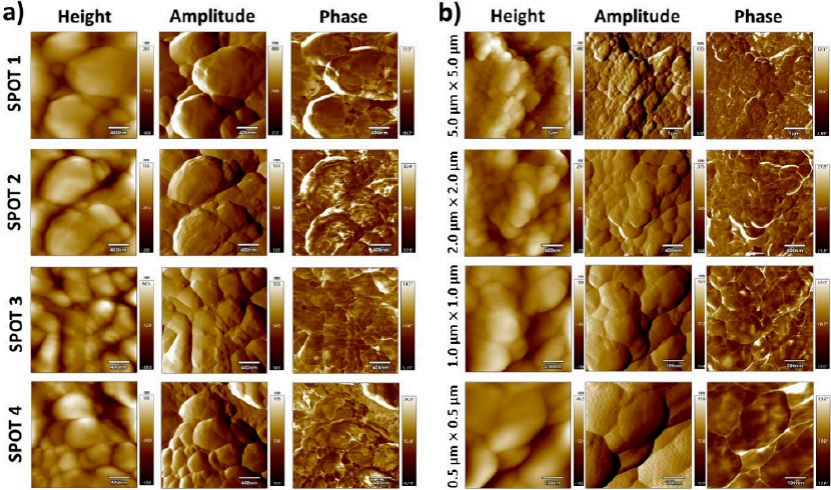
AFM imaging of the PAR 1 tablet. a) Measurements performed at four different spots over an area 2.0 μm by 2.0 μm. b) Images acquired at four different spot sizes (5.0 μm by 5.0 μm, 2.0 μm by 2.0 μm, 1.0 μm by 1.0 μm, and 0.5 μm by 0.5 μm).

Figures 1 and 2, as well as Figures S3–S6, reveal pronounced surface variations between the different PAR tablet formulations. Some PAR tablets display smooth, continuous, film-like regions, while others exhibit rougher textures with granular or plate-like domains. AFM height images resolve overall topography, AFM amplitude emphasizes edges and segregated features, and AFM phase contrast indicates compositional or mechanical heterogeneity. These differences are attributed to the choice of excipients and processing conditions, and are expected to influence critical surface-related properties, such as lubrication, wettability, and dissolution.

Similar analysis for the COMB tablets, manufactured with the same excipient make up, is presented in Figures S2, S7, and S8. These measurements indicate that variations between the COMB tablets still exist, but are less pronounced than those observed for PAR tablets. This suggests that, although not identical, the COMB tablet surfaces are more consistent overall, reflecting the use of standardized excipients and controlled processing.

### 3.2 The Application of ToF-SIMS in Pharmaceutical Tablet Surface and Subsurface Analysis

ToF-SIMS is a versatile surface analysis technique that enables the simultaneous acquisition of wide *m*/*z* range spectra (0–12 kDa), chemical images, and depth profiles from the same area, making it useful for the formulation development, troubleshooting, and quality control of tablets. ToF-SIMS produces singly charged secondary ions. Because tablet matrices are chemically complex and fragmentation during ion bombardment is extensive, spectral interferences are frequent, and peaks such as [M]^+^, [M + H]^+^, [M]^−^, or [M–H]^−^ (M stands for the parent molecule) cannot always be reliably assigned to a single component, since APIs and excipients may generate identical fragments or the mass resolution (mass resolving power) is not high enough to separate certain signals.

On the other hand, ToF-SIMS is probably best applied as a comparative tool to localize APIs, evaluate distribution heterogeneity, and detect contaminants. The method offers several advantages, including the ability to perform retrospective analysis of the acquired data set, the capability to operate in both positive and negative ion polarities, and region-of-interest evaluation that links chemical information to defined surface areas. In combination with GCIB sputtering, it allows the subsurface chemically nondestructive depth profiling of organic materials. Compared to Orbitrap-based SIMS,^11^ which provides significantly higher mass resolution, conventional ToF-SIMS offers significantly better lateral resolution and acquisition speed, making it potentially suitable for routine pharmaceutical studies. Its value is further enhanced when combined with complementary surface techniques, such as XPS for elemental and bonding information, or AFM and 3D profilometry for topography and depth calibration, which together provide a broader picture of the tablet’s surface composition and structure.

However, ToF-SIMS (and XPS, as discussed below) is not intended for high-throughput screening of pharmaceutical tablets because samples must be introduced into ultrahigh vacuum, requiring pump-down and pressure stabilization before analysis, which introduces a substantial lead time. For pharmaceutical tablets, this step can be further extended by outgassing of volatile components. In practice, this limitation can be mitigated by using an additional prechamber connected to the introduction chamber, allowing multiple samples to outgas in parallel before transfer to the analysis chamber.

ToF-SIMS is not suited to absolute or semiquantitative concentration determination because secondary-ion yields are strongly matrix dependent. For quantification, XPS can be employed (as shown below).

#### 3.2.1 Approaches to Strengthen Peak Assignment for APIs Using ToF-SIMS

Because peak assignments in ToF-SIMS are often uncertain, a systematic procedure is required to increase confidence in the signals chosen, for example, regarding subsequent ToF-SIMS imaging. In this work, five criteria were applied. First, fragment ions were tracked that can be explained by plausible fragmentation pathways and cross-checked against spectra with sufficient mass accuracy. Second, tandem MS (Section 3.2.2) was used to confirm the identity of selected fragments by inducing precursor ion dissociation and confirming structural relationships. Third, MVSA, particularly multivariate curve resolution (MCR, Section 3.2.3), was employed to extract spectral components resembling pure APIs from the complex tablet matrix. Fourth, the ToF-SIMS signals of the tablets were confirmed against those obtained from pressed reference standards of individual APIs. Finally, the spatial colocalization of the signals in the 2D and 3D imaging of the tablets was checked to ensure that multiple fragments attributed to an API originate from the same physical region of the sample. This workflow was followed in the present study to define reliable markers for the investigated molecules.

It should be noted, however, that reported ToF-SIMS fragmentation mechanisms are available for only a limited number of compounds, which remains a challenge for confident signal assignment.

#### 3.2.2 Challenges in ToF-SIMS Spectra Measured on Pharmaceutical Tablets

The ToF-SIMS spectra of pharmaceutical tablets and pure APIs are inherently complex, as illustrated in Figure 3 and Figure S9, because both API(s) and excipients contribute to a large number of fragment ions across the analyzed mass range. This complicates peak assignment and increases the risk of misinterpretation, particularly in cases where different compounds generate secondary ions at very similar or the same *m*/*z* values. Reliable interpretation requires familiarity with fragmentation mechanisms and careful consideration of possible interferences, which can limit routine application. The specialized expertise needed, combined with the relatively small number of instruments available (compared to other analytical techniques), explains why ToF-SIMS is still less commonly applied in pharmaceutical analysis despite its unique capabilities as regards molecular-level surface characterization.

**Figure 3:**
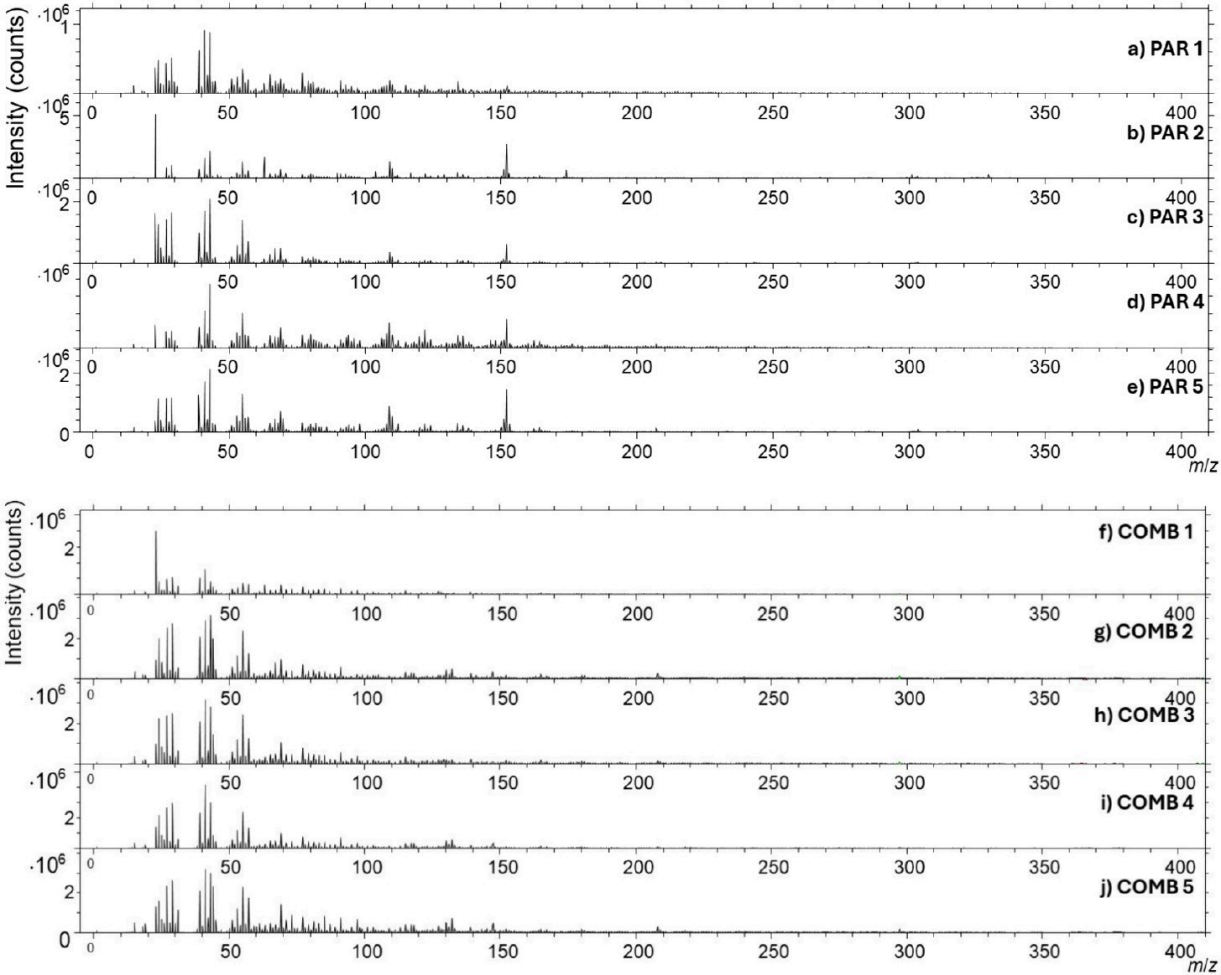
Wide *m*/*z* range ToF-SIMS spectra for the a) PAR 1, b) PAR 2, c) PAR 3, d) PAR 4, e) PAR 5, f) COMB 1, g) COMB 2, h) COMB 3, (i) COMB 4, and j) COMB 5 tablets. Spectra were acquired over a 500 μm by 500 μm area using the all-purpose analyzer mode and spectrometry LMIG mode with an acquisition time of 180 s.

Figure 3 presents the wide *m*/*z* range ToF-SIMS spectra obtained from different PAR and COMB tablets, illustrating the complexity of the spectra arising from the presence of API(s) and excipients. Such complexity makes the technique difficult for many potential users in the analytical field to interpret, discouraging it as a routine method, as the extraction of useful information requires careful spectral treatment and validation. The spectra were collected using the so-called all-purpose analyzer mode and spectrometry LMIG mode (these modes were named by IONTOF). The all-purpose analyzer mode is the standard ToF-SIMS acquisition setting that balances mass resolution and surface sensitivity. The spectrometry LMIG mode employs the primary ion gun optimized for mass spectral acquisition. It delivers a focused, pulsed primary ion beam that enhances ion yield and mass resolution, making it suitable for detailed spectral analysis before switching to imaging modes.

In order to improve confidence in peak assignment, the reference standards of PAR, IND, AMLO, and PER were pressed into tablet form and analyzed. Both MS^1^ (Figure S9) and MS^2^ (Figures 4 and S10) ToF-SIMS spectra were acquired after sputtering the surface with a 5 keV Ar_2000_^+^ at a target current of 1 nA for 10 min to remove adventitious carbonaceous species that possibly adsorbed from the atmosphere and to minimize potential cross-contamination from the previous tablet pressing process (however, the pressing device was thoroughly cleaned before the next use). This approach reduced or completely eliminated the influence of surface impurities on the ToF-SIMS spectra.

**Figure 4:**
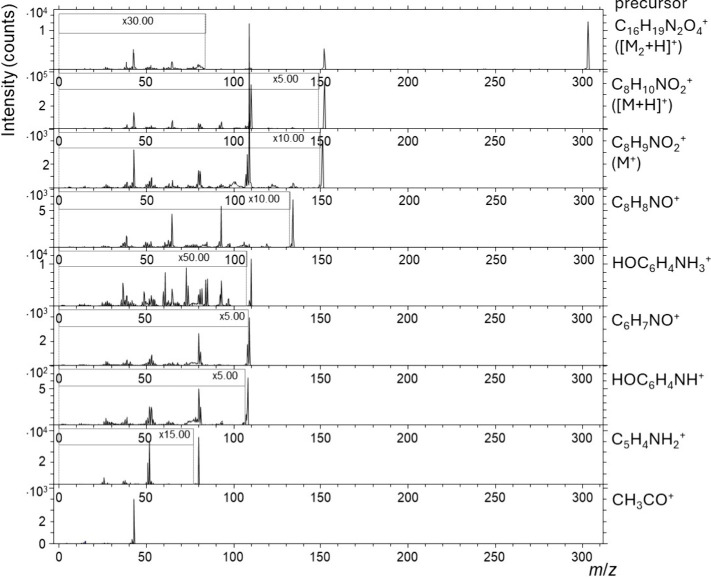
MS^2^ ToF-SIMS spectra for different precursor ions measured on the pressed tablet of the PAR reference standard. The spectra were acquired on a 200 μm by 200 μm area.

Tandem ToF-ToF SIMS (MS^2^) improves structural confidence by isolating a precursor ion and prompting high-energy collision-induced dissociation (CID, which is >1.2 keV) in a gas cell (He, in the present case, while the use of Ar or N_2_ is also possible). The resulting product-ion spectrum provides fragments that help discriminate isobars and corroborate assignments made from the MS^1^ spectrum. However, the achievable mass resolving power is limited. The mass resolution of the precursor ion selection and product ion is typically approximately 3000 (or lower) and 1000 (or lower), respectively. Consequently, some spectral interferences can remain, and chemical formula confirmation is weaker than with Orbitrap-based MS^2^ SIMS, which offers substantially higher mass resolution and mass accuracy. ToF-ToF MS^2^ spectra acquisition also takes longer than MS^1^ because only a narrow precursor window is transmitted, and because fragmentation lowers ion counts, which prolongs the accumulation time to reach an acceptable signal-to-noise ratio. Practical transmission efficiency is typically around 70%, with additional losses present when narrow timing gates are employed.

An example of such an analysis is presented in Figures 4 and S10, where MS^2^ ToF-SIMS spectra were acquired for precursor ions representative of a particular API. The MS^2^ ToF-SIMS spectra in Figure 4 (PAR reference standard) and Figure S10 (IND, AMLO, PER reference standards) can confirm the targeted structures of the organics. By selecting a defined precursor and measuring its high-energy CID fragments, the MS^2^ data can establish specific product ions that are difficult to ascribe to excipients, thereby reducing false positives from MS^1^ alone. Practically, these MS^2^ fingerprints enable a stricter marker selection by retaining only MS^1^ ions for imaging whose precursors generate mechanism-consistent product ions in the reference standard. Even with the known limitations of ToF-ToF SIMS (due to lower mass resolution), the combination of specific fragments and isotope signatures yields sufficient selectivity to confirm imaging markers. Despite these advantages, a meaningful interpretation of MS^2^ ToF-SIMS spectra still requires a strong understanding of organic fragmentation pathways. Without such expertise, the additional data provides limited analytical value.^12^

#### 3.2.3 Use of MVSA Methods in Combination with ToF-SIMS

MVSA is increasingly applied to ToF-SIMS data to handle the complexity of multicomponent surfaces. It helps to reveal key differences within or between samples, to locate specific compounds, and to confirm surface modifications. Principal component analysis (PCA) is the most widely used MVSA approach and often serves as the starting point for applying other advanced methods to interpret ToF-SIMS data sets. MCR is used to uncover the chemical composition of complex samples, producing ToF-SIMS data that are easier to interpret. The method generates a set of MCR factors, with each MCR factor corresponding to a deconvoluted chemical feature within the mixture. MCR can be applied to all ToF-SIMS outputs, including spectra, images, and depth profiles.^11b,13^ Moreover, machine learning is increasingly being applied to ToF-SIMS data evaluation, as it offers automated ways to recognize patterns in large and complex data sets. Machine learning is likely to become a central part of ToF-SIMS data processing in the near future.^14^

In this work, unique signals are extracted using MCR. A unique signal is a ToF-SIMS peak that, after MCR processing, contributes exclusively (or most significantly) to the single MCR factor representing the target component and not to other MCR factors. Its image colocalizes with the score map of that MCR factor and thus serves as a specific marker in this data set. In positive polarity, MCR successfully extracted unique signals for each API (only the markers with *m*/*z* higher than 120 were retained for further analysis). For PAR, the unique signal was C_8_H_10_NO_2_^+^ at *m*/*z* 152.07. IND was represented by several unique signals, including *m*/*z* 132.07 (C_9_H_10_N^+^), 147.09 (C_9_H_11_N_2_^+^), 217.97 (C_7_H_5_ClNO_3_S^+^), 365.06 (C_16_H_16_ClN_3_O_3_S^+^), 366.07 (C_16_H_17_ClN_3_O_3_S^+^), and 388.05 (C_16_H_16_ClN_3_O_3_SNa^+^). AMLO was identified by unique signals at *m*/*z* 297.14 (C_14_H_21_N_2_O_5_^+^), 407.14 (C_20_H_24_N_2_O_5_Cl^+^), 408.15 (C_20_H_25_N_2_O_5_Cl^+^), 409.15 (C_20_H_26_N_2_O_5_Cl^+^), and 431.13 (C_20_H_25_N_2_O_5_ClNa^+^). Lastly, PER was distinguished by a unique signal at *m*/*z* 172.13 (C_9_H_18_NO_2_^+^). These ions, isolated through MCR, provided markers for subsequent 2D and 3D ToF-SIMS imaging of the tablets.

An example of the effectiveness of this approach is shown in Figure 5, where the MCR score 3D image and the corresponding 3D ToF-SIMS image display good agreement. This match demonstrates that MCR extracts mathematically independent components and recovers chemically meaningful distributions. The analytical value lies in improving confidence that the chosen signals originate from the APIs of interest rather than from excipients or artifacts. Moreover, such a correlation between the MCR score 3D image and the raw ToF-SIMS 3D image is essential in assigning peaks, validating imaging markers, and interpreting the API distribution within multicomponent tablets.

**Figure 5:**
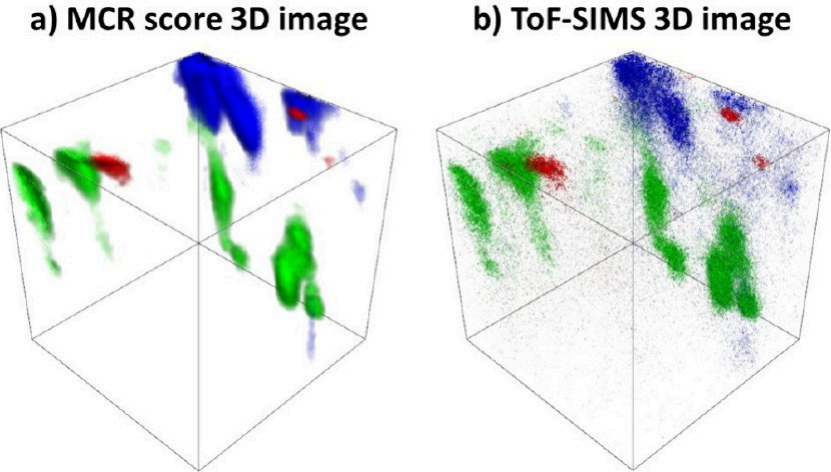
a) The MCR score 3D image for MCR factors representing IND (red), AMLO (green), and PER (blue), and b) the 3D ToF-SIMS image representing unique signals for IND (red), AMLO (green), and PER (blue). The ToF-SIMS data were obtained in the same manner as for Figure 7.

#### 3.2.4 Data Correction and Postprocessing in ToF-SIMS Imaging

One common artifact that can be present in ToF-SIMS analysis is the shadowing effect, which can arise with sputtering into subsurface regions. Surface roughness or embedded particles can shield underlying areas from the sputter beam, leading to distorted depth profiles.

Postprocessing of ToF-SIMS data is crucial for obtaining reliable chemical images from pharmaceutical tablets. For 3D imaging, several corrections can be applied. Mass shifts may occur during sputtering due to variations in surface potential, which result from encountering subsurface regions of differing chemical composition. In this work, all 3D images were corrected for such effects using dedicated software (SurfaceLab 7.5).

Another limitation can be the differential sputter rate between organic API(s) and excipients, and inorganic excipients such as SiO_2_. Inorganics are less efficiently removed by GCIB sputtering, which can distort depth scales and result in sputter-induced roughening. This is shown in Figure S1, where some areas are sputtered at a faster rate compared to other areas. Moreover, similar is shown in Figure 6, where the sputter depth was shallower for the PAR tablets containing SiO_2_ (the PAR 1, PAR 3, and PAR 5 tablets), while the PAR tablets without this inorganic excipient had deeper craters (the PAR 2 and PAR 4 tablets).

**Figure 6:**
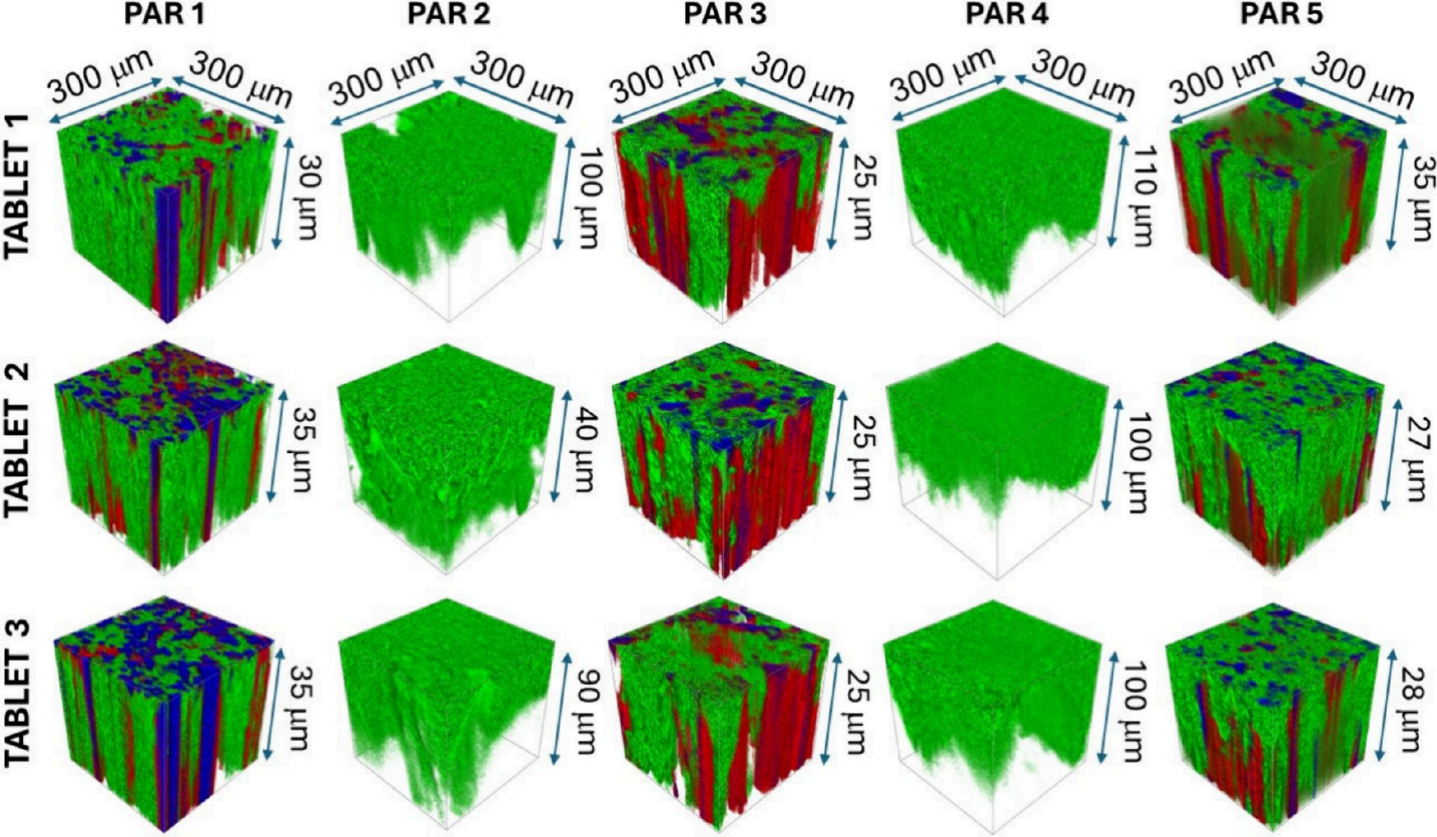
3D ToF-SIMS images of the PAR tablets; green: the signal at *m*/*z* 152.07 for C_8_H_10_NO_2_^+^, representing PAR; red: the signal at *m*/*z* 23.98 for Mg^+^ originating from Mg stearate and talc (for the PAR 1, PAR 3, and PAR 5 tablets); and blue: the signal at *m*/*z* 27.98 for Si^+^ originating from SiO_2_ and/or talc (shown for the PAR 1, PAR 3, and PAR 5 tablets). Note that Mg- and Si-containing species were absent in the PAR 2 and PAR 4 tablets. The depth (*z*-axis) is indicated (as determined using 3D profilometry).

Surface topography also complicates interpretation as the software generates 3D images under the assumption of a flat surface. Unless scanning probe microscopy (SPM) is integrated with ToF-SIMS, the initial roughness cannot be accounted for, whereas ToF-SIMS/AFM hybrid systems can measure and correct the same area prior to analysis. Unfortunately, such instrumentation is currently very rare.

For 2D imaging, lateral drift during long acquisitions can blur images due to primary beam instability. Therefore, lateral shift correction (a software correction method) was applied herein, which is particularly important for 2D imaging using delayed extraction analyzer and fast imaging LMIG modes, where acquisition times are longer (Section 3.2.6).

Additionally, advanced ToF correction is required to compensate for distortions caused by surface topography. On strongly topographic surfaces, the extraction field becomes distorted, and the local acceleration field varies across pixels; secondary ions emitted from different pixels then enter the analyzer with different kinetic energies, which worsens mass resolution and can distort ion images. In order to address this, an advanced ToF correction was used that recalculates flight time on a per-pixel basis prior to image reconstruction, improving the mass resolving power and spatial reliability. This correction was implemented for all 2D images in the present study.

The above-mentioned corrections ensure that both spatial and chemical information are represented as exactly as possible.

#### 3.2.5 Determination of the API Spatial Distribution by ToF-SIMS

A ToF-SIMS 3D imaging experiment is performed by alternating analysis and sputtering cycles. The analysis beam generates secondary ions, while the sputter beam gradually removes material to create a crater. Repeated measurements at the crater center build a ToF-SIMS depth profile or 3D ToF-SIMS image until the desired depth is reached. In the present case, a 10 keV Ar_2000_^+^ sputter GCIB was used. The crater depth, determined by 3D profilometry, is then used to calibrate the sputter time axis (*z*-axis), allowing the construction of depth profiles and 3D chemical maps of selected ions.

Figures 6, 7, and S11 illustrate how this approach reveals the spatial distribution of APIs and certain excipients within different tablet formulations. In the COMB tablets (Figures 7 and S11), ToF-SIMS shows that PER, AMLO, IND, and certain excipients are not uniformly distributed but display local heterogeneity across the sampled volume. This finding has practical consequences. If APIs are not evenly dispersed, splitting a tablet may not yield two halves with equivalent dosages, which raises questions about the dose uniformity in such formulations. In contrast, the PAR tablets (Figure 6) generally exhibit a more consistent distribution of the major API, although Mg- and Si-containing excipients also appear as distinct, segregated domains.

**Figure 7:**
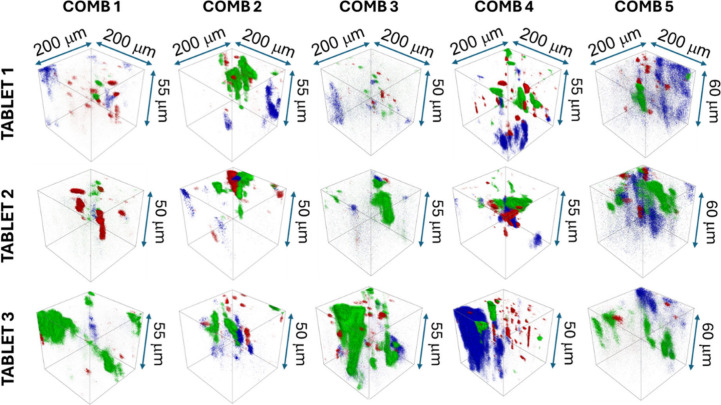
3D ToF-SIMS images obtained from the COMB tablets showing the distribution of PER (blue), AMLO (green), and IND (red) (for every API, the unique signals were used as explained in Section 3.2.3). The depth (*z*-axis) is indicated (as determined using 3D profilometry).

ToF-SIMS 3D imaging, therefore, provides a direct means of evaluating the effectiveness of API miscibility and distribution in pharmaceutical formulations at the submicron scale. Among the available analytical techniques, ToF-SIMS uniquely combines molecular specificity with submicron (or lower) spatial resolution, enabling 3D visualization of the APIs and excipients. However, interpretation requires caution. Rough tablet surfaces, as shown by 3D profilometry and AFM, can distort the apparent depth and distribution of signals due to shadowing and variable sputter rates. As discussed above, software typically assumes a flat initial surface, so topography-induced artifacts can compromise exact interpretation. For this reason, the 3D ToF-SIMS images herein should be regarded as informative rather than strictly exact. However, in the present case (Figures 6, 7, and S11), topography effects do not alter the overall impression of API distribution, and the information obtained from such imaging is very useful.

These results demonstrate that ToF-SIMS is a powerful tool for assessing the spatial distribution of APIs and excipients within solid dosage forms. It enables the recognition of inhomogeneities, the evaluation of formulation consistency, and the assessment of whether processing achieves the intended uniformity, thereby providing valuable insights relevant to quality control and formulation development.

#### 3.2.6 High Lateral Resolution 2D ToF-SIMS Imaging of the Topographic Samples

Delayed extraction analyzer and fast imaging LMIG modes can be employed to obtain high lateral resolution chemical maps of APIs and excipients on tablet surfaces. In delayed extraction, ions are accelerated into the analyzer with a controlled time lag after sputtering, which can yield mass resolution above 10000 for the signals of interest. This mode provides images with both high lateral and mass resolution over small regions (herein 52 μm by 52 μm, at 512 pixels by 512 pixels), making it especially valuable for distinguishing APIs from excipients on the submicron scale. An additional advantage is its improved performance on topographic samples, since the delayed extraction compensates for variations in the initial ion energy caused by surface roughness, thereby reducing image distortion. The disadvantages of this mode are a reduced secondary ion yield, partial loss of low *m*/*z* species during the delay, and longer acquisition times, often several hours per image. Fast imaging mode, in contrast, sacrifices some mass resolution to accelerate data collection, allowing for high lateral resolution imaging (in this case, approximately 100 nm lateral resolution). Such high lateral resolution (together with the simultaneous acquisition of molecule-specific information) is not achievable with conventional Raman or FTIR spectroscopy. SEM/EDXS imaging can deliver even higher lateral resolution, but it is restricted to elemental information, rather than molecular.

An example of such 2D imaging is presented in Figure 8, where the signal at *m*/*z* 152.07 (C_8_H_10_NO_2_^+^) originates from PAR, while the signal at *m*/*z* 23.98 (Mg^+^) reflects contributions from Mg stearate and/or talc. The signal at *m*/*z* 27.98 (Si^+^) derives from SiO_2_ and/or talc. For the PAR 5 tablet, the additional fragment at *m*/*z* 127.05 (C_6_H_7_O_3_^+^) is characteristic of saccharides and here is attributed to the maize starch excipient, which generates this ion from glucose units in the polymer backbone.^15^ The signals in Figure 8 demonstrate how delayed extraction analyzer mode and fast imaging LMIG mode can localize API- and excipient-derived ions within a single HR data set.

**Figure 8:**
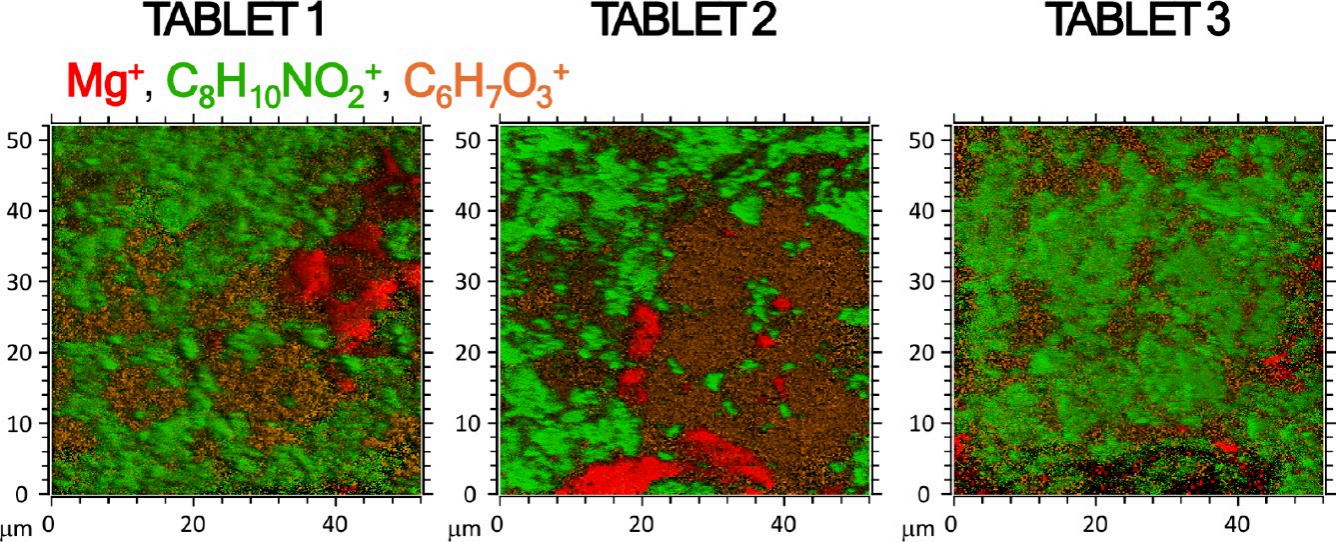
2D ToF-SIMS images obtained on three different PAR 5 tablets measured in a delayed extraction analyzer and fast imaging LMIG modes to acquire high-lateral resolution ToF-SIMS images by using signals with relatively high mass resolution.

Although the long acquisition times exceed the static SIMS dose (often considered in the range of 10^12^–10^13^ ions · cm^–2^), this is acceptable for the present objective. Features substantially thicker than a monolayer are targeted, so limited ion-induced alteration of the outermost molecules is not expected to affect spatial distribution maps. The higher dose is used to improve counting statistics and image reliability (edge definition, low intensity markers), which is prioritized here. In order to avoid misinterpretation, undisturbed monolayer chemistry in this case is not assumed, and image evaluation should be performed on a relative basis.

#### 3.2.7 Large-Area 2D ToF-SIMS Imaging Using ToF-SIMS

ToF-SIMS is typically restricted to a maximum field of view of 500 μm by 500 μm, which limits direct visualization of larger tablet regions. To overcome this, a stitching approach was applied, in which multiple adjacent image tiles are measured and computationally combined to reconstruct a continuous, large-area chemical map. This method extends the accessible imaging field while retaining the spatial resolution (≤3 μm in the present case) of each tile.

An example is provided in Figure 9 for the PAR 5 and COMB 3 tablets. For PAR 5, the relatively flat surface enabled imaging of a 3.0 mm by 3.0 mm area, whereas for COMB 3, only a 1.0 mm by 1.0 mm area could be imaged because the rounded surface caused focus and alignment limitations. The unique marker signals defined in Section 3.2.3 were used to extract the distributions of IND, AMLO, and PER in the COMB 3 tablet, whereas the signal for C_8_H_10_NO_2_^+^ was employed to represent PAR in the PAR 5 tablet.

**Figure 9:**
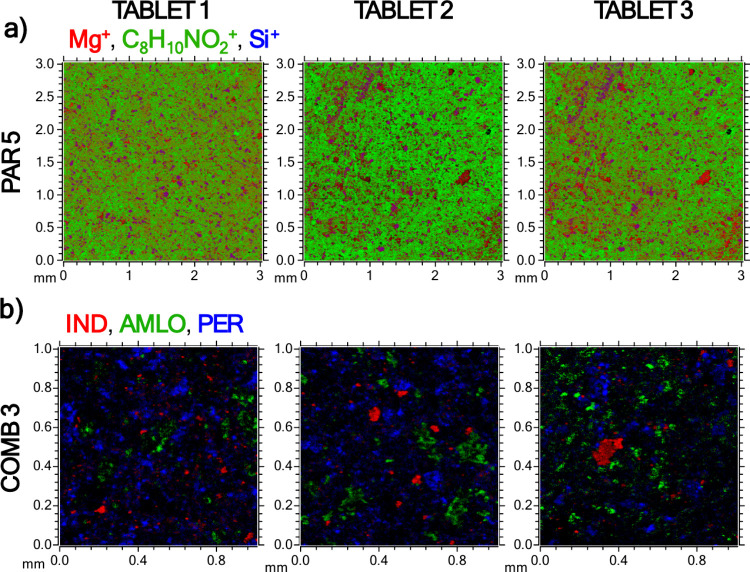
Large-area 2D ToF-SIMS images obtained by stitching. a) Three different PAR 5 tablets, showing the distribution of PAR (green), the Mg^+^ signal (red), and the Si^+^ signal (blue) over a 3.0 mm by 3.0 mm area; b) three different COMB 3 tablets, imaged over a 1.0 mm by 1.0 mm area, with the API distribution visualized using unique signals for IND (red), AMLO (green), and PER (blue).

The ToF-SIMS large-area 2D images demonstrate the spatial variation of APIs and excipients across different regions. For instance, in the PAR 5 tablet, PAR appears broadly distributed, while excipient domains remain locally concentrated. In the COMB 3 tablet, the three APIs exhibit distinct yet overlapping spatial patterns, indicating a heterogeneous mixing of components. Comparing three separate measurement spots further highlights how composition can vary within a single tablet, reflecting differences in formulation processing and local microstructure.

Analytically, such large-area 2D imaging provides valuable insights into API homogeneity, excipient localization, and overall formulation quality. It allows an assessment of whether the API and excipient domains are uniformly dispersed or segregated, which is directly relevant to dosage uniformity, dissolution behavior, and manufacturing reproducibility.

A further practical advantage for such large-area imaging, where very high lateral resolution is not required, is that millimeter-scale fields can be acquired within a few minutes under suitable conditions. In contrast, obtaining a 3.0 by 3.0 mm Raman or FTIR chemical image generally requires substantially longer acquisition times because the data are typically collected by point-by-point mapping.

### 3.3 The Application of XPS for Pharmaceutical Tablet Surface and Subsurface Analysis

Although some studies have demonstrated its usefulness, XPS remains underutilized for pharmaceutical tablet analysis. Some examples of its applications include XPS analyses of loratadine,^16^ ciprofloxacin,^17^ sildenafil,^18^ PAR,^19^ ibuprofen,^20^ and dipyrone,^21^ with early work further highlighting its potential for systematic surface and subsurface characterization in this field.^22^ XPS provides (semi)­quantitative information on elemental composition and can reveal chemical states and bonding types. XPS analysis probes only the outer few nanometers of a surface. Combining survey and HR spectra with depth profiling using GCIB can reveal both the presence of APIs and excipients and how their chemical environments change between the surface and subsurface. For pharmaceutical tablets, this is valuable for assessing excipient distribution, surface segregation, and possible surface modifications that influence stability, dissolution, and performance.

Caution is required when interpreting XPS data, as misassignments are common. XPS is better regarded as a confirmation technique rather than a primary identification tool for specific functional groups or molecules. Overfitting (deconvolution) of XPS spectra is a common issue, and assignments of specific chemical states often carry uncertainty. In the absence of clear spectral features or corroboration from complementary techniques, additional deconvoluted peaks should not be introduced arbitrarily, as this can lead to serious misinterpretation.^23^ However, combining ToF-SIMS and XPS is advantageous in order to confirm the presence of specific species on the sample surface. While XPS is highly element-specific, some spectral interferences may occur. These are usually resolved by examining alternative photoelectron XPS peaks of the same element. Unlike ToF-SIMS, which can detect analytes down to the ppb or even ppt range, the detection limit of XPS is higher, typically around 0.1 at. %.

The depth in XPS depth profiling is generally lower than in ToF-SIMS depth profiling because XPS spectra acquisition requires significantly longer acquisition times. In practice, XPS spectra are measured only at selected sputter intervals, whereas ToF-SIMS acquires spectra continuously during each sputter cycle, typically in the 100–200 μs range. However, the GCIB technology is identical for both XPS and ToF-SIMS, allowing comparable sputter depths to be accessed.^24^ In this work, for the PAR tablets, sputtering was restricted to shallower depths due to the higher PAR content relative to the APIs in the COMB tablets. In contrast, longer sputtering times were applied for the COMB tablets to capture depth-dependent variations within a single formulation and to enable comparisons across different COMB tablets.

#### 3.3.1 Elemental and Chemical State Analysis of the PAR Tablets by XPS

An example of XPS analysis for pharmaceutical tablets is given in Figure 10. The origin of signals for certain elements can arise from different sources. The lowest spectra in Figure 10 for particular PAR tablets (color coding is indicated in the figure) represent the surface before sputtering. In the case of the PAR tablets, the C 1s signal arises from PAR and organic excipients. However, adventitious carbonaceous species that might be adsorbed on the surface can also contribute to the C 1s signal for the outermost position. The dashed lines at 1, 2, and 3 in Figure 10 most likely represent C–C/C–H (284.8 eV), C–O (at approximately 286.5 eV), and C=O/COO (at 288.0–289.0 eV) environments, respectively. The O 1s signal originates from PAR and excipients, with two distinctly different O-environments designated at dashed lines 1 and 2. The signal for N 1s primarily originates from PAR. In contrast, for the PAR 2 and PAR 3 tablets, the N 1s signal can also originate from povidone. The Na 1s signal, detected in PAR 2 and PAR 3, originates from sodium carboxymethyl starch. The Si 2p signals in PAR 1, PAR 3, and PAR 5 arise from talc and/or SiO_2_, both of the declared excipients. The Mg 2p signal in PAR 1, PAR 3, and PAR 5 comes from Mg stearate, with an additional contribution from talc. This example demonstrates that all elements expected from the PAR tablet formulations are detected, highlighting the strength of XPS in confirming elemental composition and showing that these species are already present at the outermost surface. Ca-containing species in the tablets were not reported in the manufacturer’s specifications for any PAR tablet formulation. However, nonintense Ca 2p peaks were detected in some survey spectra (Figure S12, for PAR 3, and also for PAR 1 and PAR 5), suggesting low amounts likely introduced during processing or originating from minor undeclared impurities (the Ca^+^ signal was also detected using ToF-SIMS). Although potassium sorbate was listed by the manufacturer as an excipient in the PAR 5 tablet, no K-related signal was detected by XPS within the analyzed area and sputter depth. In contrast, ToF-SIMS measurements revealed the presence of K^+^.

**Figure 10:**
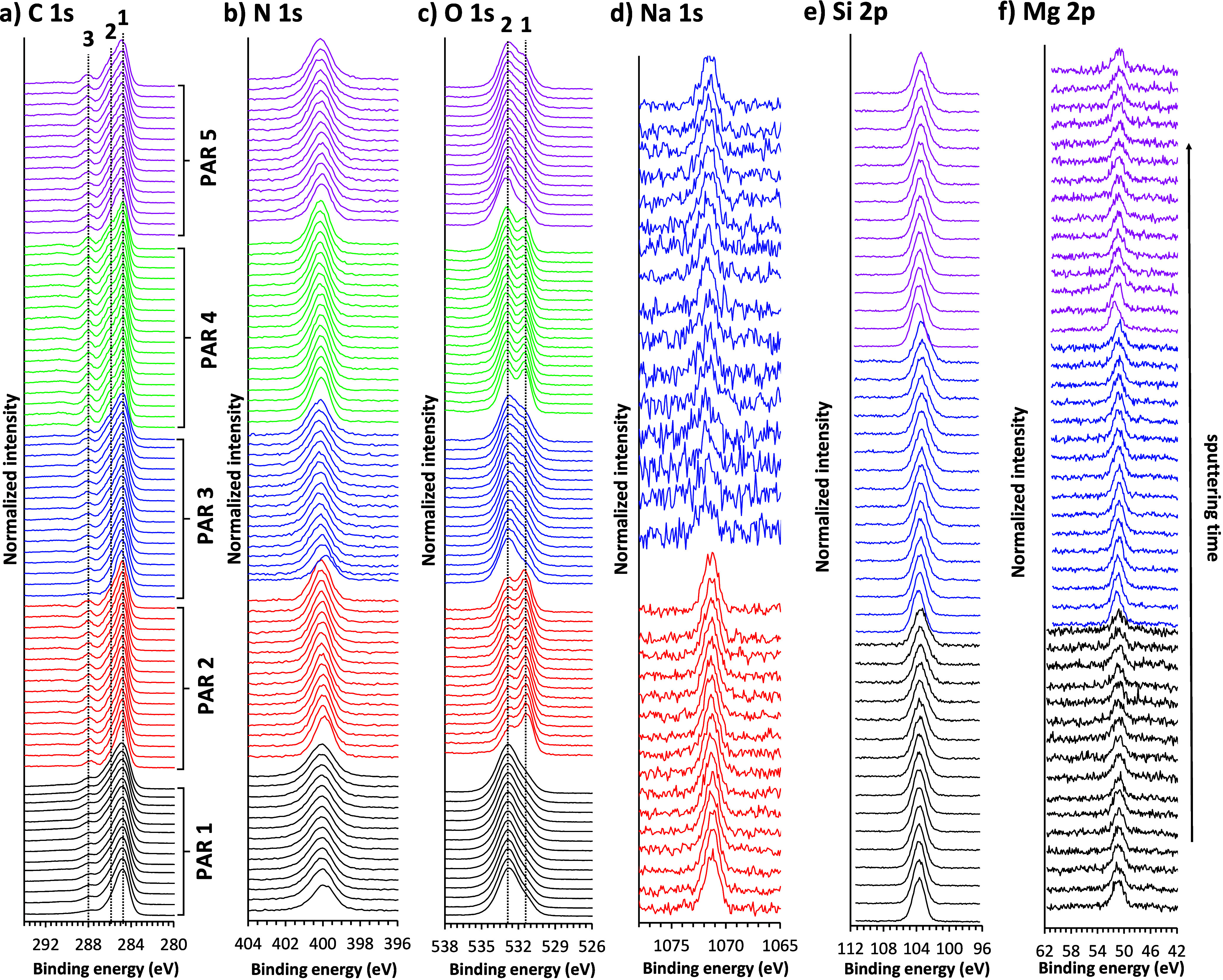
HR XPS spectra for a) C 1s, b) N 1s, c) O 1s, d) Na 1s, e) Si 2p, and f) Mg 2p measured during depth profiling using 10 keV Ar_1000_^+^ (the lowest spectra represent measurements before sputtering). Color codes in different spectra are the same for a given PAR tablet as designated in a).

Moreover, XPS can determine elemental composition at depth. Figure 10a shows the C 1s spectra obtained during GCIB depth profiling, where sputtering enhances the contributions assigned to C–O and C=O/COO in PAR 1 and PAR 3, whereas in PAR 5 this increase is observed for the C=O/COO component. In contrast, the C 1s spectra of PAR 2 and PAR 4 remained essentially unchanged with sputtering. In Figure 10b (N 1s spectra), the lowest spectra (before sputtering) for the PAR 1, PAR 2, and PAR 3 tablets are shifted to more negative binding energies relative to the spectra after the first sputtering cycle, indicating a change from surface-affected environments to bulk-like environments. This can reflect different PAR binding with excipients. Furthermore, Figure 10c shows that the O 1s spectra for the PAR 2 and PAR 4 tablets have a more intense, more negative binding energy component at dashed line 1 than that at dashed line 2. Upon sputtering these two PAR tablets, the contribution at dashed line 2 in the O 1s spectra increased relative to that at dashed line 1, suggesting a different O-environment in the subsurface region compared to that in the surface. In contrast, the PAR 3 and PAR 5 tablets exhibit the opposite trend, with the dashed line 1 component becoming more intense relative to the dashed line 2 spectral feature upon sputtering. For the PAR 1 tablet, no significant O 1s spectral changes are observed at depth.

In contrast, the Na 1s, Si 2p, and Mg 2p spectra show minor depth dependence (Figures 10d–f). The main exception is the Mg 2p spectrum in PAR 5, where the Mg 2p spectrum before sputtering is shifted to a more positive binding energy than the spectra measured after sputtering. This likely reflects a distinct surface microenvironment and/or contributions from two Mg-bearing excipients (Mg stearate and talc) at the outermost layer. No significant Mg 2p peak shift is observed for PAR 1 or PAR 3. For the Si 2p spectra, PAR 3 and PAR 5 also display a slight positive binding-energy shift in the spectrum before sputtering relative to deeper layers, consistent with a different surface SiO_2_/talc environment, which is removed by sputtering. These observations indicate subtly different surfaces compared with the subsurface environments for Mg- and Si-containing excipients, whereas the Na 1s spectra remain essentially unchanged at depth for the PAR 2 and PAR 3 tablets.

The above demonstrates the potential of XPS for identifying formulation-related issues, particularly cases where API-excipient interactions are undesirable or require detailed investigation.

#### 3.3.2 XPS Depth Profiling for the PAR Tablets

Depth profiling converts XPS spectra into quantitative elemental trends as a function of sputter time (or sputter depth, if the crater depth is independently measured, e.g., by 3D profilometry), allowing a direct comparison of the five PAR tablet formulations. In such measurements, a focused sputter beam is used to remove material layer by layer, creating a sputter crater from which spectra are acquired. Care must be taken to ensure that only the flat central region of the crater is analyzed, as the inclusion of crater edges can distort the results and lead to misinterpretation. This consideration is often overlooked but is essential to obtaining reliable depth profiles.

Figure S13 shows that the C concentration decreases after the first sputter cycle for all PAR tablets, which might signify the removal of surface adventitious carbonaceous species contamination. However, no such drop is present for PAR 2 (Figure S13b). Higher concentrations of Si and Mg are observed on the surfaces of the PAR 1, PAR 3, and PAR 5 tablets (Figure S13a,c,e), which decrease progressively with sputtering, reflecting contributions from SiO_2_/talc and Mg stearate. These elements were absent in PAR 2 and PAR 4, which is in agreement with their excipient composition. Na was determined in the PAR 2 and PAR 3 tablets and remains nearly constant at depth, originating from sodium carboxymethyl starch. The N concentration in general increases after sputtering (however, the change is not significant), reflecting the removal of adventitious carbonaceous species and/or a higher PAR surface concentration (or povidone in the case of the PAR 2, PAR 3, PAR 4, and PAR 5 tablets) in the subsurface regions.

These depth profiles confirm the declared excipient make up, demonstrate differences in surface versus bulk composition, and provide insight into formulation-specific characteristics that can explain why the PAR 2 and PAR 4 tablets differ from the PAR 1, PAR 3, and PAR 5 tablets in both XPS and ToF-SIMS analyses.

In this study, the depth profiles obtained by XPS for the PAR tablets probe shallower regions than those accessible by ToF-SIMS. However, XPS offers complementary value by providing quantitative elemental concentrations, while ToF-SIMS mainly reveals the spatial distribution of individual species.

#### 3.3.3 XPS Analysis of the COMB Tablets

In the COMB tablets, the origins of the detected elements can be assigned to both APIs and excipients (Figures S14–S18). The C 1s signal arises from APIs and excipients (cellulose, maize starch, sodium carboxymethyl starch, and Mg stearate). The O 1s signal originates from both organic groups in the APIs and excipients, as well as the inorganic species, including SiO_2_ and carbonates. Contributions to the N 1s signal are linked to all three APIs (IND, AMLO, and PER). The S 2p signal is characteristic of IND due to its sulfonamide group. The Cl 2p signal originates from chlorine-containing moieties in AMLO and IND, as well as from CaCl_2_. The Ca 2p signal is associated with CaCl_2_, while the Na 1s signal arises from sodium hydrogen carbonate and sodium carboxymethyl starch. Inorganic excipients contribute to the Si 2p signal (from SiO_2_) and the Mg 2p signal (from Mg stearate).

The C 1s spectra of the COMB tablets can be interpreted using the same assignments as for the PAR tablets, with dashed lines 1, 2, and 3 (Figures S14b–S18b). Upon sputtering, the ratio between these contributions changes, shifting intensity from the spectral feature at dashed line 1 to dashed line 2. This trend reflects the removal of surface organics and adventitious carbonaceous species, revealing subsurface C environments more representative of excipients and APIs, or changes in the relative composition and interactions between APIs and excipients at depth.

Among all elements, the O 1s environment shows the most pronounced formulation-dependent variation in the COMB tablets (Figures S14b–S18b). With sputtering, these relative contributions evolve differently depending on the tablet. The diverse O sources in the formulations can explain this behavior. Furthermore, at the surface, O environments can be influenced by the segregation of lubricants (e.g., Mg stearate). In addition, the outermost O-containing species may interact differently with neighboring components than those embedded deeper in the tablet, further contributing to the observed variation.

For N-, S-, and Cl-containing species, changes at depth are also evident. The N 1s peak is present for the spectra representing the surface for all COMB tablets and decreases after the initial sputtering steps (S14d–S18d). This, for all COMB tablets, indicates a relative surface enrichment of N-bearing APIs and lower concentrations in the immediate subsurface. However, it does not necessarily imply absence in deeper regions, but rather that levels fall near or below the detection limit. Similarly, the S 2p signal from IND decreases with sputter time (Figures S14e–S18e). Although the overall API content increases from COMB 1 to COMB 5, the N concentration at the outermost surface does not follow this trend (Figures S19b–S23b).

The Cl 2p peak (which can represent IND, AMLO, and CaCl_2_) is detected at the outermost surface (with the lowest spectra in Figures S14f–S18f) and shifts in binding energy with sputtering. Such spectral change can come from two effects, i.e., a varying mixture of Cl sources (surface-enriched CaCl_2_ versus API-bound Cl) and/or depth-dependent modifications in the microenvironment of the chlorinated APIs. Ca 2p is the most intense before sputtering and drops at depth­(Figures S14g–S18g), indicating a substantial CaCl_2_ contribution to the initial Cl 2p signal for the outermost position. However, S 2p (for IND) is also the most intense at the surface, confirming the presence of IND and contributing to the Cl 2p signal. As sputtering progresses and the CaCl_2_ contribution diminishes (evident from the Ca 2p signal drop), the Cl 2p signal becomes increasingly dominated by API-bound Cl, and its position reflects different API-excipient interactions in the subsurface relative to the outermost layer.

The Na 1s signal increases with sputtering for all COMB tablets (Figures S19b–S23b). This suggests that, in most formulations, Na-containing excipients are more prominent in the subsurface region than at the outermost surface. In contrast, the Si 2p (Figures S14i–S18i) and Mg 2p (Figures S14j–S18j) signals generally decrease with sputtering across all COMB tablets (Figures S19b–S23b), indicating that SiO_2_ and/or talc and/or Mg stearate excipients are enriched at or near the outermost surface rather than deeper in the tablet.

After the sputtering of the COMB tablets, the crater depth was measured by 3D profilometry, and the corresponding average sputter rate was approximately 10 nm/min.

The depth profiles in Figures S19–S23 provide quantitative trends in the elemental concentrations as a function of sputter depth, allowing for a direct comparison of the five COMB tablet formulations. This data reveals how elements from APIs and excipients are distributed between the surface and subsurface. Differences among formulations are evident. It is shown that some have surface enrichment of species such as Mg, Si, or Ca, while others display more uniform depth profiles. In particular, N, S, and Cl depth profiles highlight formulation-specific differences in the API distribution and excipient interactions. These profiles illustrate how varying excipient content and manufacturing choices lead to distinct surface and subsurface compositions across the COMB tablets.

In conclusion, XPS provides an analytical value for tablets by quantifying elemental composition and revealing chemical states within the outer 5–10 nm layer, as well as by providing further depth when combined with GCIB sputtering, which enables depth-resolved characterization. It confirms excipient-related signals (SiO_2_/talc, Mg stearate, CaCl_2_, Na salts) and API-related elements (N, S, Cl), identifies surface enrichment or depletion, and can detect processing residues and surface modifications.

The combination of XPS and ToF-SIMS analyses can therefore support, for example, assessments of dose uniformity, lubrication, coating adhesion, dissolution risk, stability, and batch comparability. The scarcity of such studies likely reflects a limited expertise in conducting them. Standardized workflows and paired ToF-SIMS/XPS protocols can lower this barrier and broaden routine use in pharmaceutical tablet analysis.

## 4 Conclusions

This study presents a comprehensive surface analysis workflow for solid pharmaceutical tablets, integrating time-of-flight secondary ion mass spectrometry (ToF-SIMS), X-ray photoelectron spectroscopy (XPS), atomic force microscopy (AFM), and 3D profilometry. Two case studies were intentionally selected to bracket analytical difficulty, i.e., a high-dose single active pharmaceutical ingredient (API), paracetamol (PAR) tablets, and low-dose combination (COMB) tablets containing indapamide (IND), amlodipine (AMLO), and perindopril (PER). Five formulations were examined in each set to capture the variability in the formulations. Across all steps, the methods were deployed in a coordinated manner to extract complementary information from the same materials system at both the micro- and nanoscale levels.

AFM and 3D profilometry established the geometric context needed for chemical analysis. They provided information about surface topography from the nanometer to the millimeter scale, identified spot-to-spot heterogeneity within tablets, and measured crater depths after gas cluster ion beam (GCIB) sputtering.

ToF-SIMS provided molecularly specific mapping and depth profiling. Reference standards (powders) were pressed into the tablets and measured to confirm fragmentation behavior and to select candidate markers. Multivariate curve resolution was applied to ToF-SIMS spectra to isolate unique signals for each API. Targeted tandem (ToF-ToF) MS^2^ ToF-SIMS experiments were used on selected precursors to increase confidence in the structural relationships among the fragments. With markers defined, 2D chemical images were acquired, large fields were assembled by stitching, and GCIB depth profiles were measured to visualize subsurface distributions. Delayed extraction and fast imaging modes enabled submicrometer lateral detail while maintaining high mass resolving power. Practical postacquisition corrections were also discussed. These steps produced images and depth profiles that locate APIs and excipients, reveal surface segregation, and visualize interfacial layering that cannot be acquired from conventional analytical techniques currently used for such analysis.

XPS supplied quantitative elemental and chemical-state information for the outer few nanometers and, with GCIB sputtering, regarding the subsurface. Survey XPS spectra confirmed the expected elements from APIs and excipients and exposed low-content species that can influence processing and performance. High-resolution XPS spectra tracked changes in C, O, N, and other core levels between the surface and subsurface, indicating differences in the chemical environment that align with the heterogeneity observed by ToF-SIMS. In combination, XPS and ToF-SIMS provided depth-resolved elemental and molecular information, linking them, and proving the cross-confirmation of assignments.

Analytically, this combined workflow delivers capabilities that are difficult to achieve with conventional techniques for solid pharmaceutical tablets. It localizes APIs and excipients at the submicrometer scale in 2D. It identifies the surface enrichment of excipients and detects interfacial layers that are only a few nanometers thick. It quantifies subsurface elemental composition and captures depth-dependent changes in the chemical state. It reports geometric features that control charging, extraction fields, and ion yields. These outputs can support formulation design, troubleshooting, and batch comparison. They can also facilitate the reverse engineering of commercial products and targeted investigations into coating, compression, and storage effects.

The work also clarifies practical constraints and why surface analysis remains underused. Instruments are expensive and not widely available. Expertise is required in vacuum handling, charge control, beam alignment, crater metrology, spectral fitting, chemometrics, and image processing. Measurements are susceptible to variations in surface cleanliness, topography, and differences in sputter rates across mixed organic/inorganic matrices.

Despite these challenges, the results herein indicate that surface analysis should be adopted more widely in solid-dose development and quality investigations. A practical approach includes routine precharacterization by AFM and 3D profilometry to select regions for analysis, the use of reference standards to define ToF-SIMS markers, the application of GCIB, and the cross-confirmation of molecular maps with XPS elemental and chemical state data. When deployed in this structured manner, ToF-SIMS, XPS, AFM, and 3D profilometry provide surface-specific and depth-resolved evidence that strengthens the conclusions drawn from Raman spectroscopy, infrared spectroscopy, and other analytical techniques for the analysis of the tablet’s bulk. The present study demonstrates that these techniques provide important information that significantly enhance the understanding of the organization of APIs and excipients on the tablet surface and at depth, and therefore they merit inclusion in the analytical toolbox for modern tablet research, development, and control.

## Supplementary Material



## Data Availability

The data are available upon request.

## Notes

A summary of the supplementary 3D profilometry, AFM, ToF-SIMS, and XPS data (PDF)
