# Supplementary material for: Contemporary Techniques and Prospects in Pharmaceutical Tablet Surface Analysis
Source: ACS Meas Sci Au. 2026 Feb 2;6(2):430–44. doi: 10.1021/acsmeasuresciau.5c00178 (PMC13087966; doi:10.1021/acsmeasuresciau.5c00178)
Supplement: Supplementary file 1 [file tg5c00178_si_001.pdf]

SUPPLEMENTARY INFORMATION

Contemporary Techniques and Prospects in Pharmaceutical Tablet Surface Analysis

Matjaž Finšgar

Faculty of Chemistry and Chemical Engineering, University of Maribor, 2000 Maribor, Slovenia

S3.1 3D profilometry and AFM supporting information data

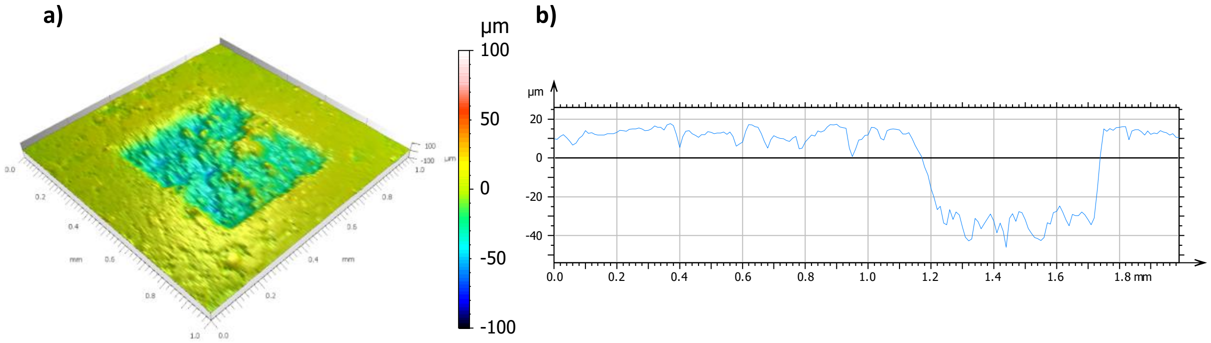

Figure S1: 3D profilometry of a sputter crater formed during ToF-SIMS depth profiling, showing a) the crater surface morphology and b) the cross-sectional profile used to assess depth and shape.

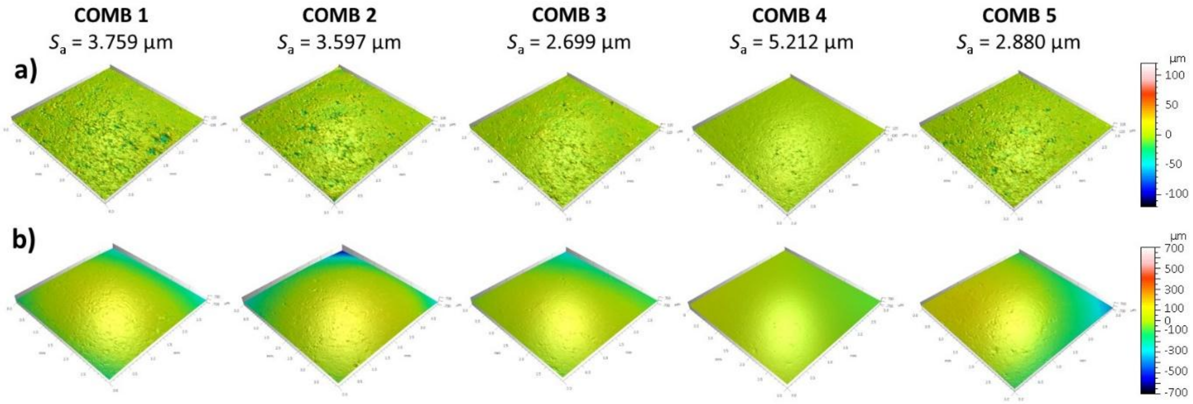

Figure S2: 3D profilometry of the COMB tablets, shown a) with and b) without form removal, with corresponding S_a values. The measurements were performed on 3.0 mm by 3.0 mm spot sizes.

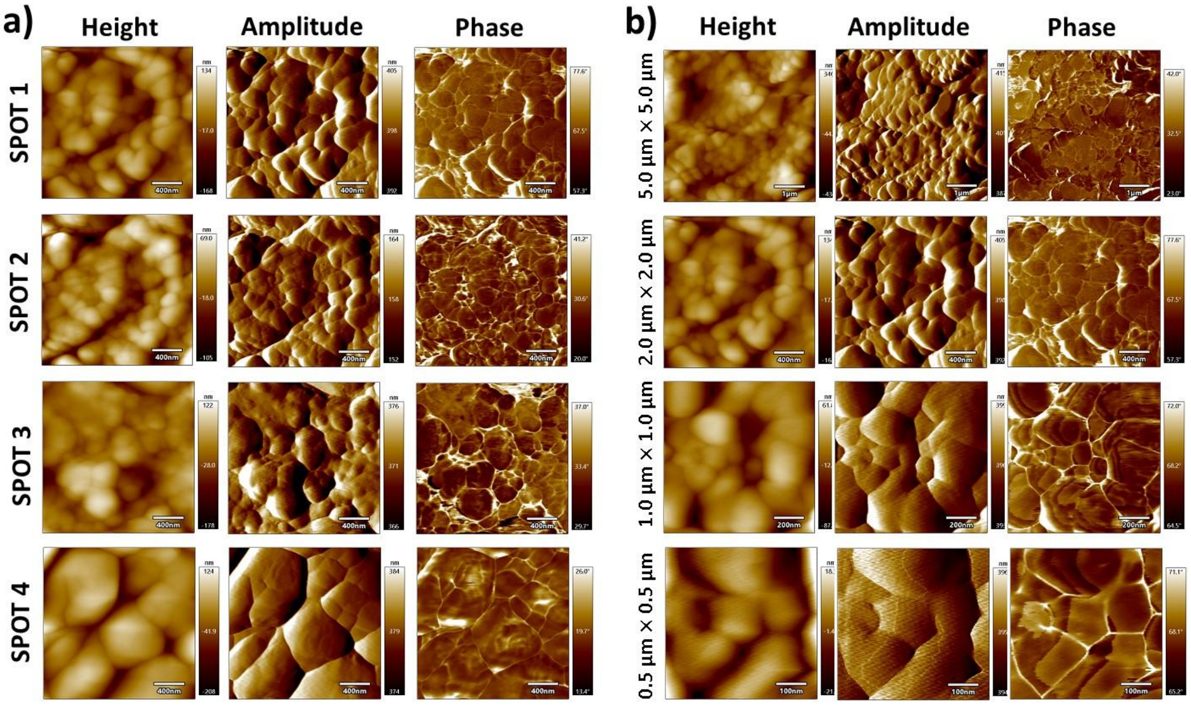

Figure S3: AFM imaging of the PAR 2 tablet. a) Measurements performed at four different spots over an area of 2.0 µm by 2.0 µm. b) Images acquired at four different spot sizes (5.0 µm by 5.0 µm, 2.0 µm by 2.0 µm, 1.0 µm by 1.0 µm, and 0.5 µm by 0.5 µm).

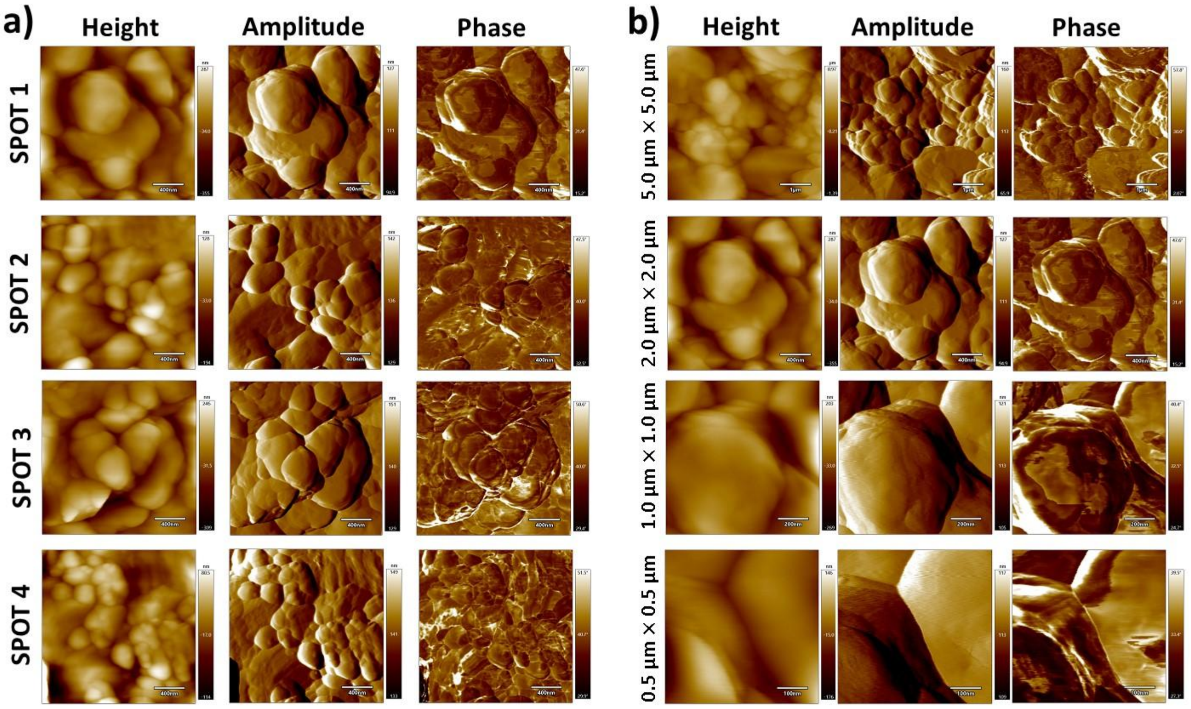

Figure S4: AFM imaging of the PAR 3 tablet. a) Measurements performed at four different spots over an area of 2.0 µm by 2.0 µm. b) Images acquired at four different spot sizes (5.0 µm by 5.0 µm, 2.0 µm by 2.0 µm, 1.0 µm by 1.0 µm, and 0.5 µm by 0.5 µm).

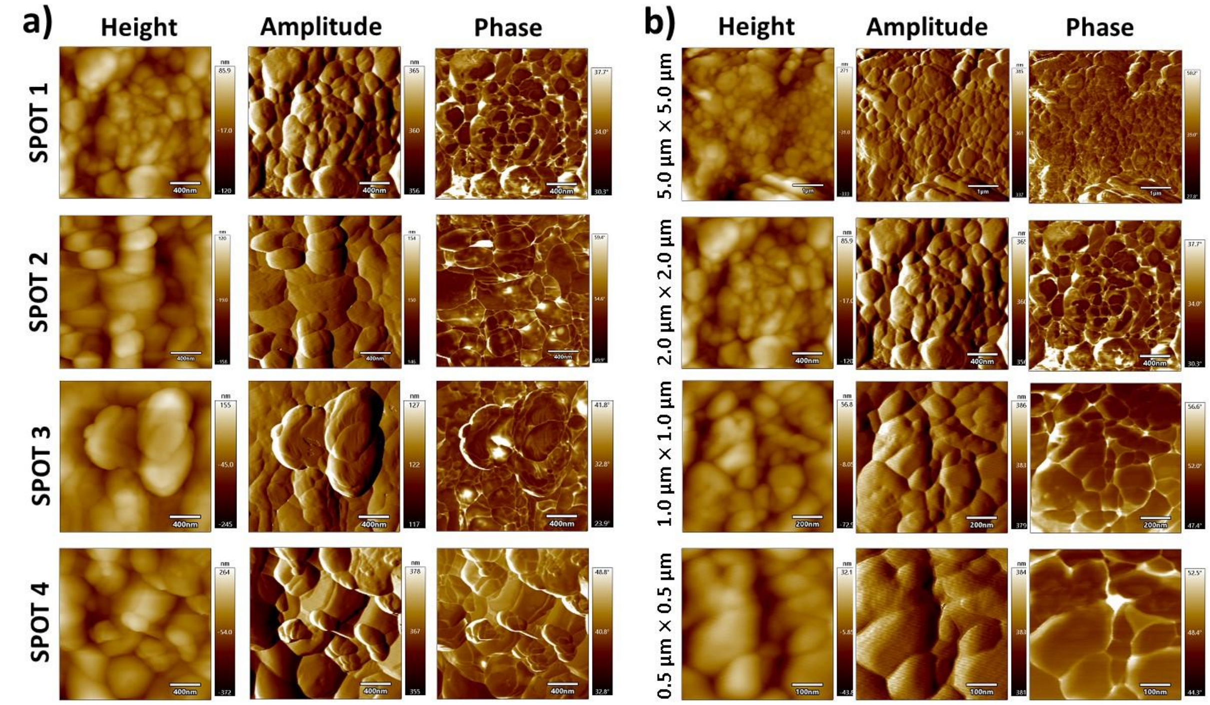

Figure S5: AFM imaging of the PAR 4 tablet. a) Measurements performed at four different spots over an area of 2.0 µm by 2.0 µm. b) Images acquired at four different spot sizes (5.0 µm by 5.0 µm, 2.0 µm by 2.0 µm, 1.0 µm by 1.0 µm, and 0.5 µm by 0.5 µm).

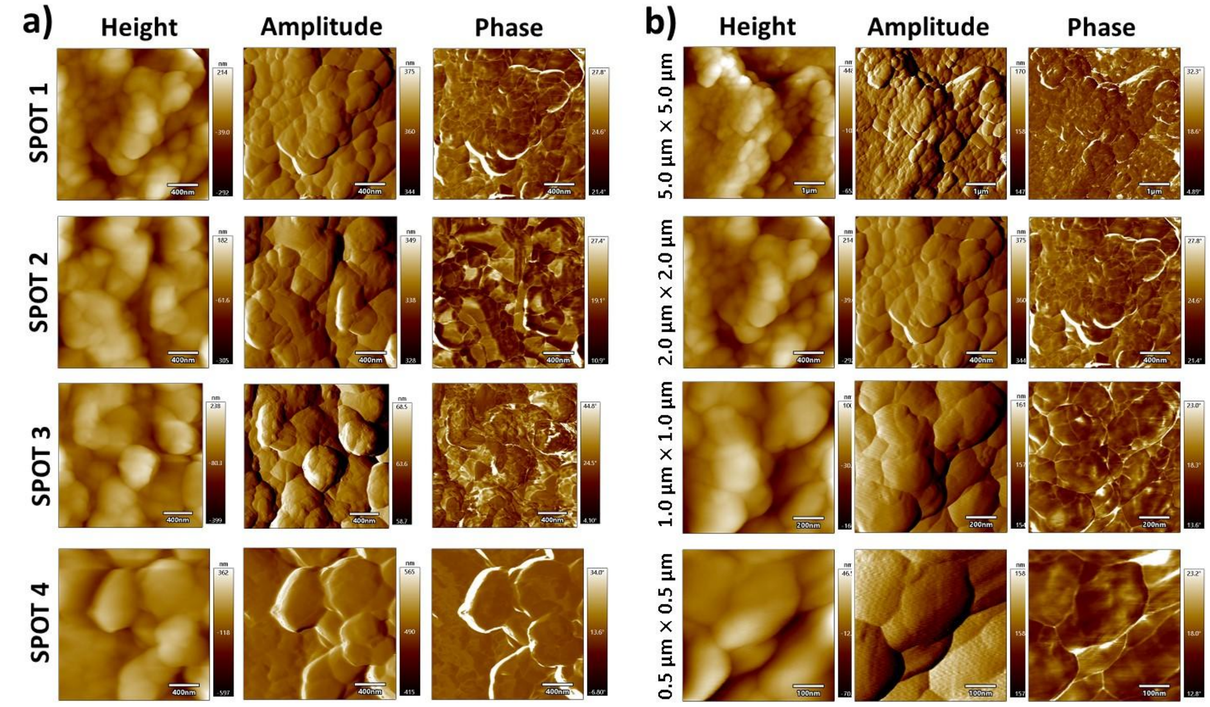

Figure S6: AFM imaging of the PAR 5 tablet. a) Measurements performed at four different spots over an area of 2.0 µm by 2.0 µm. b) Images acquired at four different spot sizes (5.0 µm by 5.0 µm, 2.0 µm by 2.0 µm, 1.0 µm by 1.0 µm, and 0.5 µm by 0.5 µm).

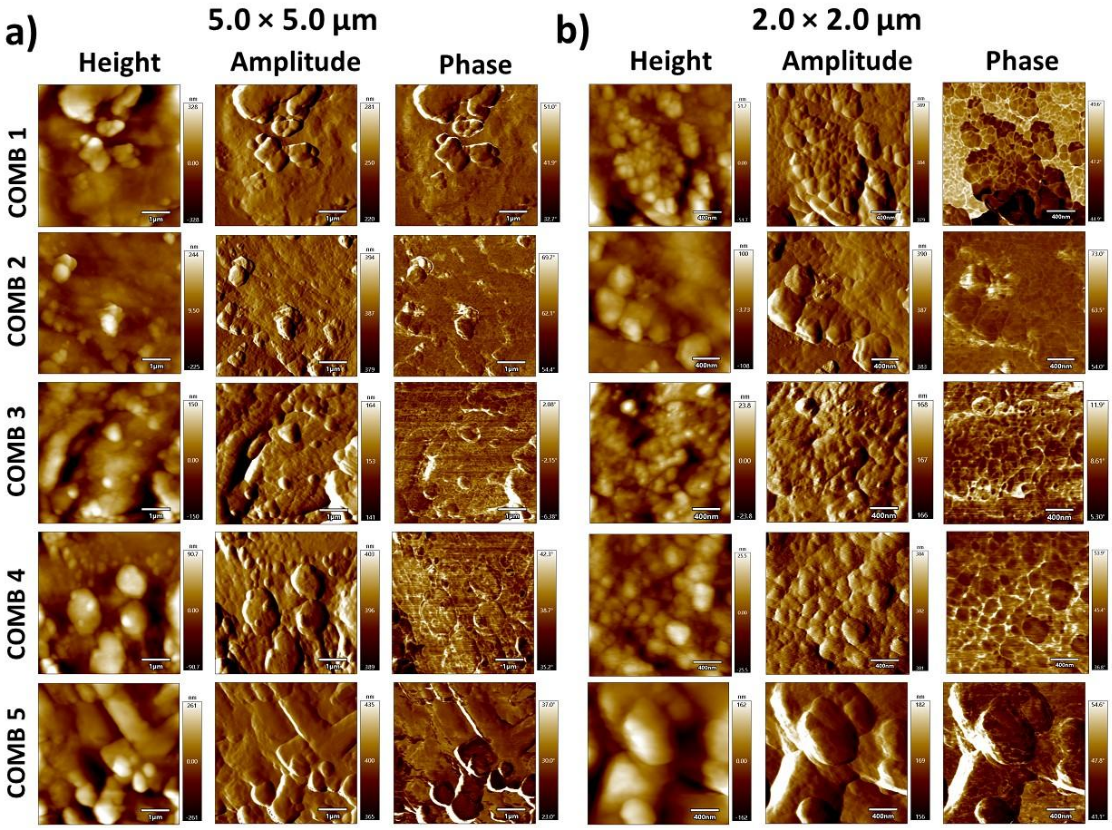

Figure S7: AFM imaging of the COMB tablets measured over an area of a) 5.0 µm by 5.0 µm and b) 2.0 µm by 2.0 µm.

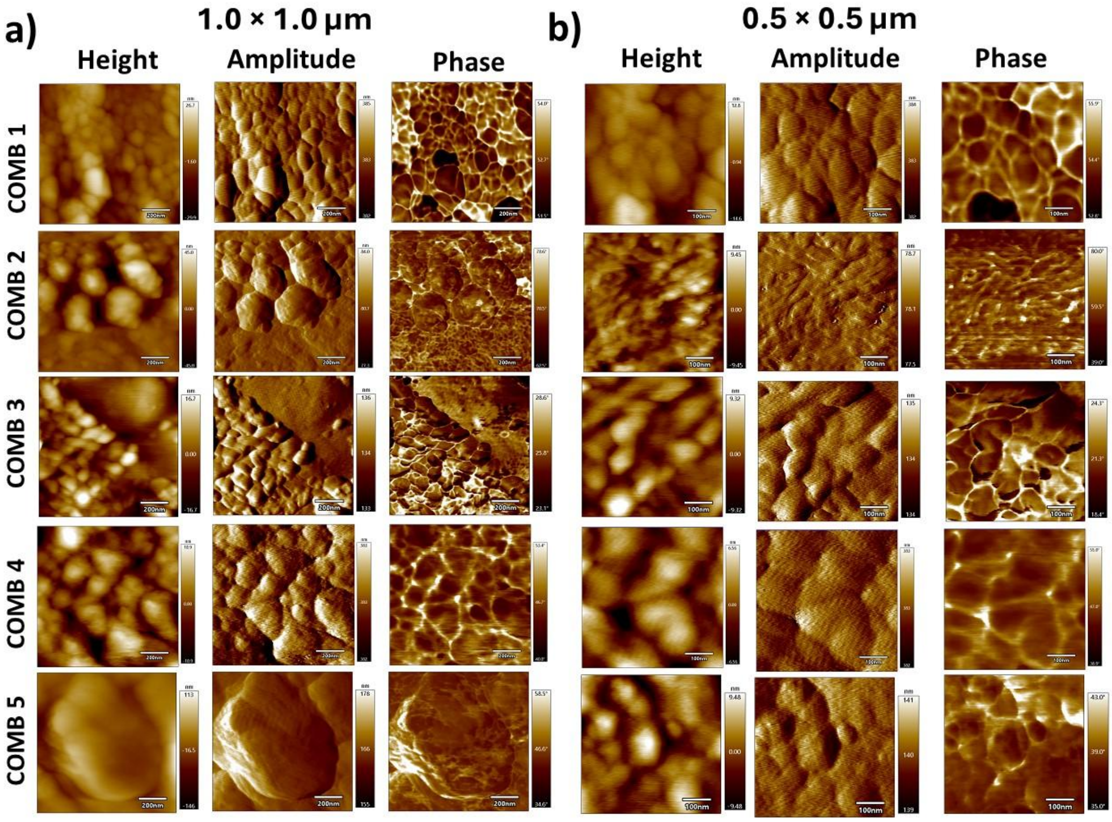

Figure S8: AFM imaging of the COMB tablets measured over an area of a) 5.0 µm by 5.0 µm, and b) 2.0 µm by 2.0 µm.

S3.2 ToF-SIMS supporting information data

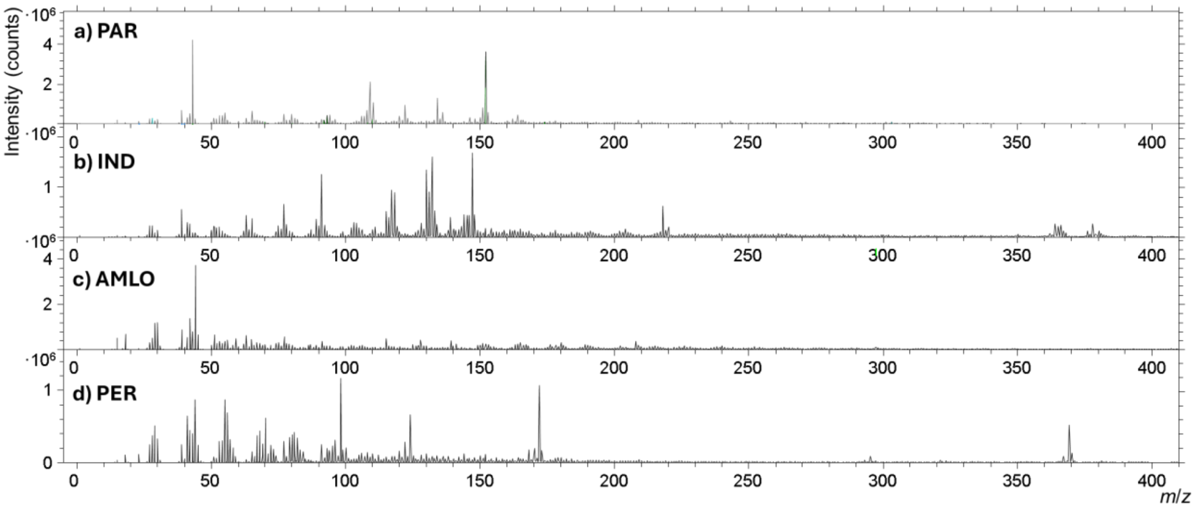

Figure S9: MS[1] spectra measured on a) PAR, b) IND, c) AMLO, and d) PER reference standards pressed into a tablet and after sputtering for 10 min using 5 keV Ar_{2000}^{+} (1 nA).

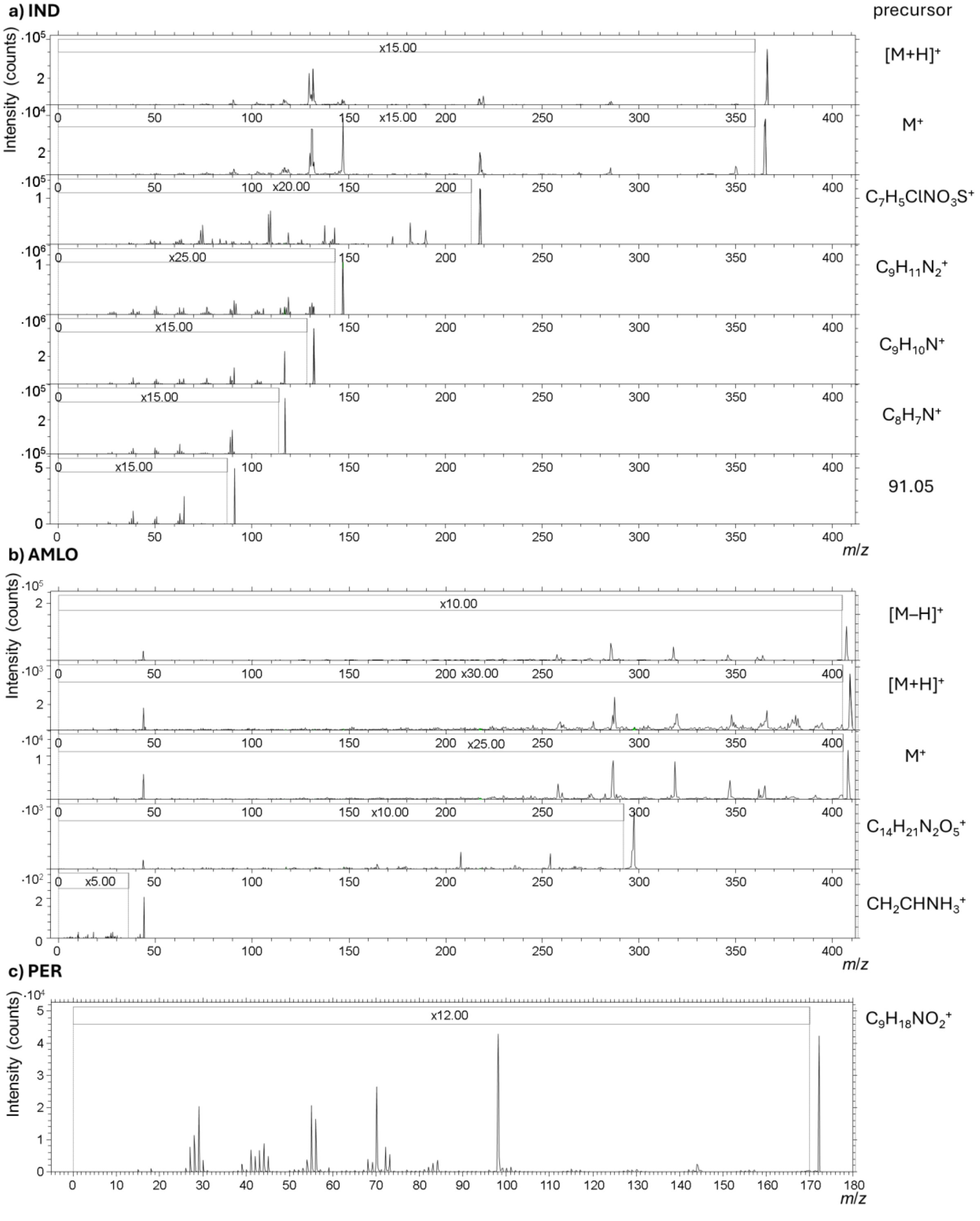

Figure S10: MS^2 spectra for selected precursor ions measured on a pressed tablet of the a) IND, b) AMLO, and c) PER reference standards after sputtering for 10 min using 5 keV Ar_{2000}^+ (1 nA).

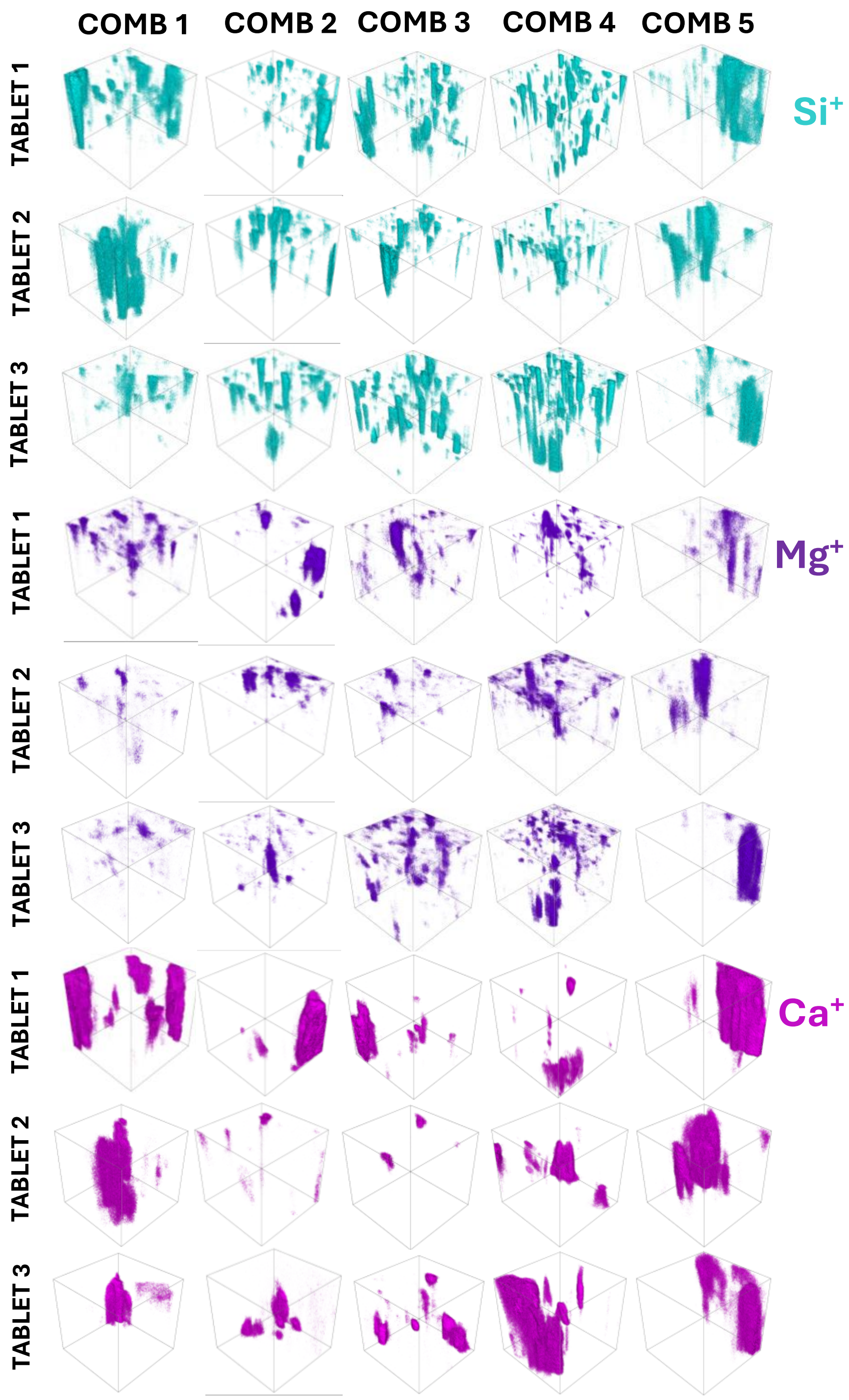

Figure S11: The spatial distribution of Si^+, Mg^+, and Ca^+ for different COMB tablet formulations obtained using ToF-SIMS by sputtering the surface with 10 keV Ar_{1000}^+. The sputter depth is the same as given in Figure 7.

S3.3 XPS supporting information data

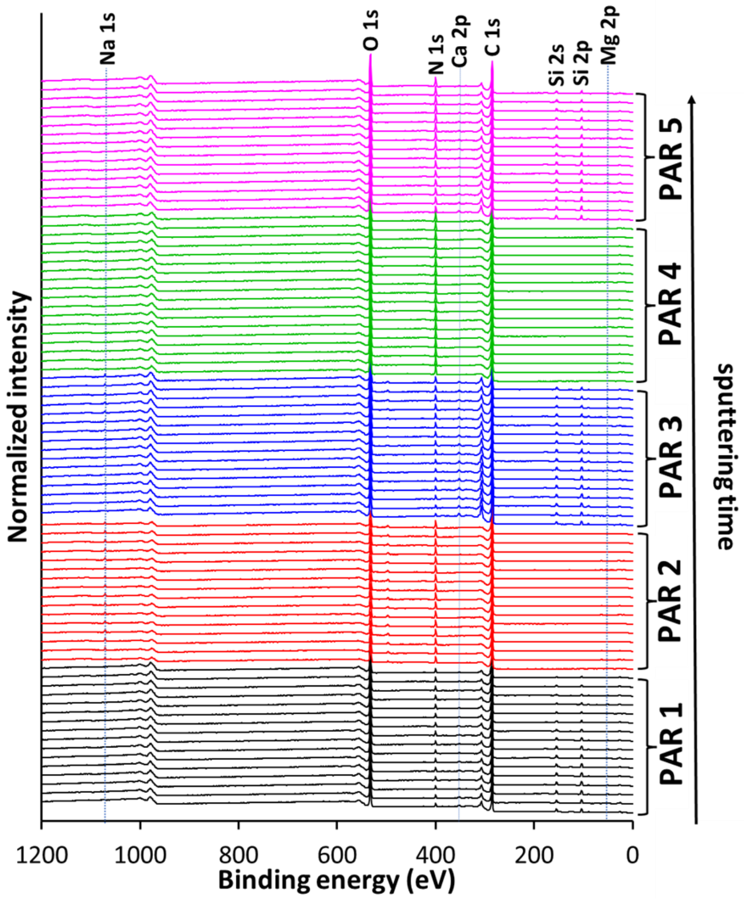

Figure S12: Survey spectra acquired during XPS depth profiling. The lowest spectrum corresponds to the surface before sputtering. Color coding for each PAR tablet follows that used in Figure 10.

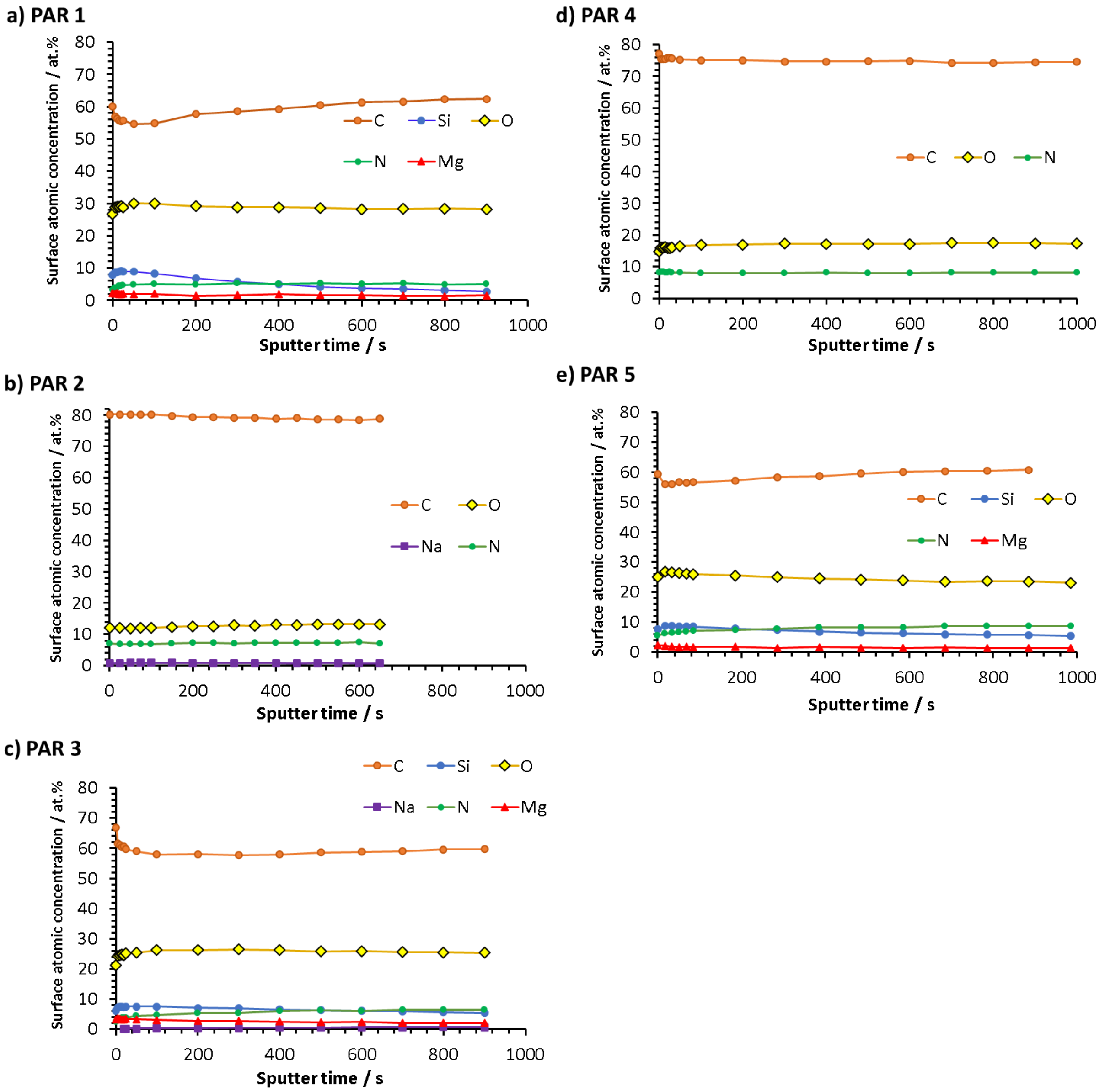

Figure S13: XPS depth profiles for different PAR tablets: a) PAR 1, b) PAR 2, c) PAR 3, d) PAR 4, and e) PAR 5.

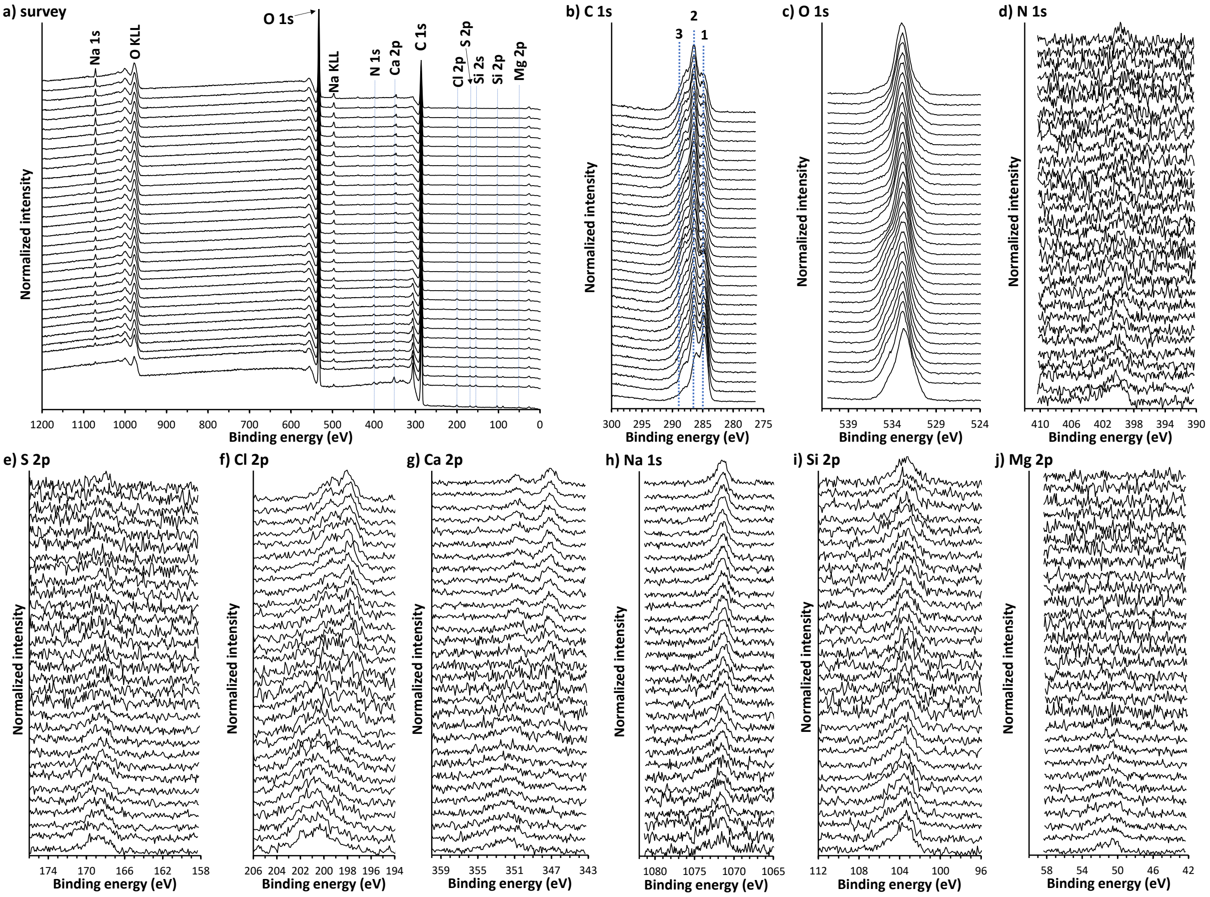

Figure S14: XPS analysis of the COMB 1 tablet: a) survey XPS spectra and HR spectra for b) C 1s, c) O 1s, d) N 1s, e) S 2p, f) Cl 2p, g) Ca 2p, h) Na 1s, i) Si 2p, and j) Mg 2p. Depth profiling was performed with a 10 keV Ar_{1000}^{+} sputter beam; the lowest spectra correspond to the surface before sputtering, with increasing depth shown from bottom to top.

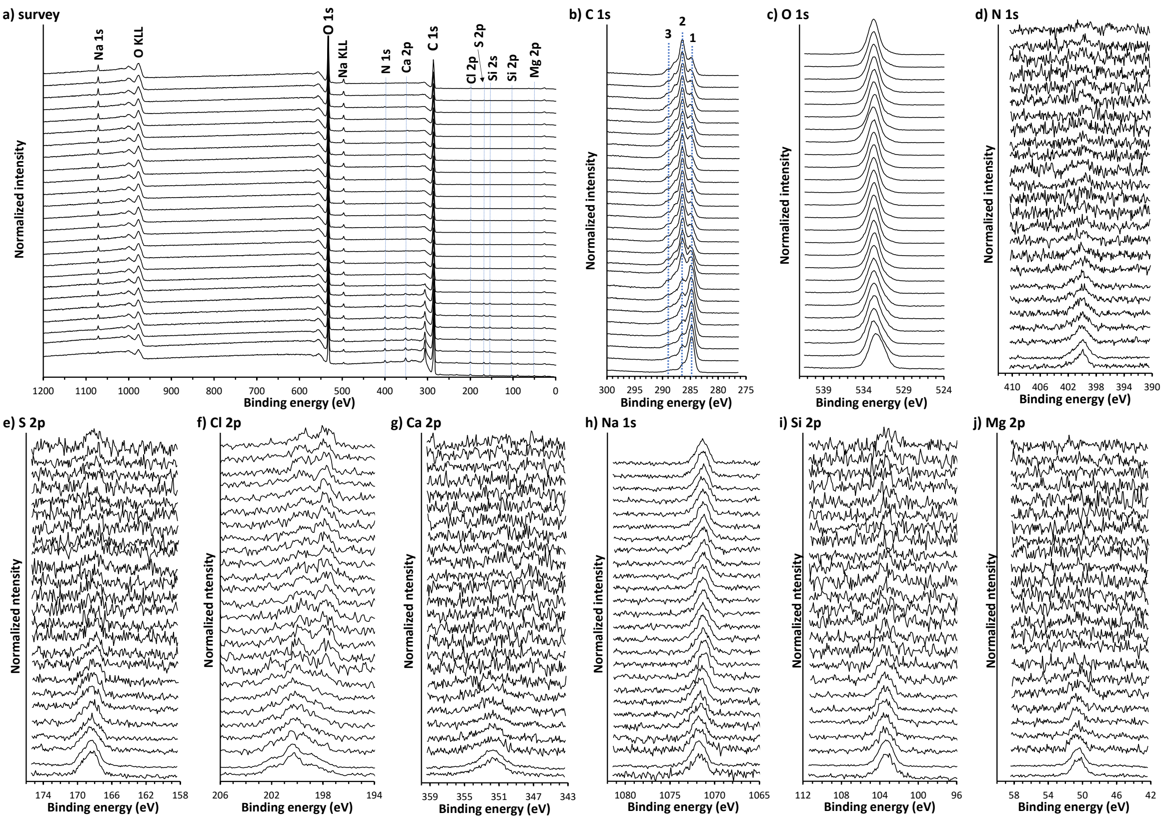

Figure S15: XPS analysis of the COMB 2 tablet: a) survey XPS spectra and HR spectra for b) C 1s, c) O 1s, d) N 1s, e) S 2p, f) Cl 2p, g) Ca 2p, h) Na 1s, i) Si 2p, and j) Mg 2p. Further details are as presented in Figure S14.

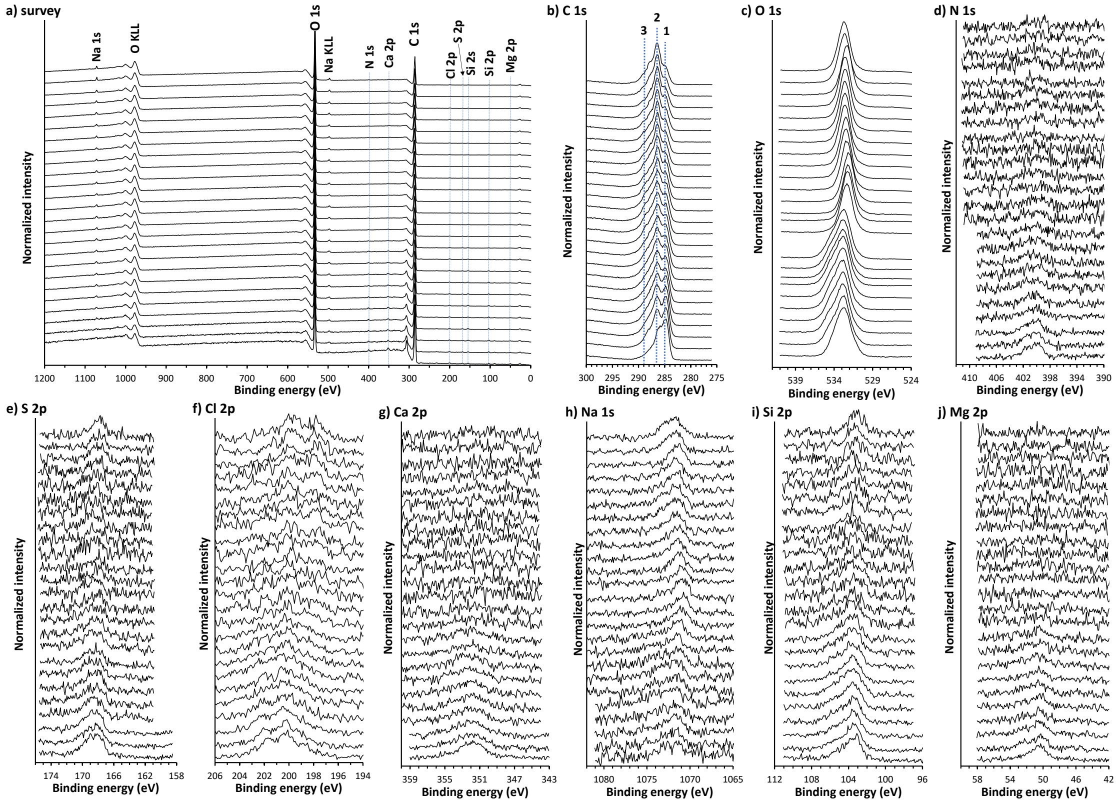

Figure S16: XPS analysis of the COMB 3 tablet: a) survey XPS spectra and HR spectra for b) C 1s, c) O 1s, d) N 1s, e) S 2p, f) Cl 2p, g) Ca 2p, h) Na 1s, i) Si 2p, and j) Mg 2p. Further details are as presented in Figure S14.

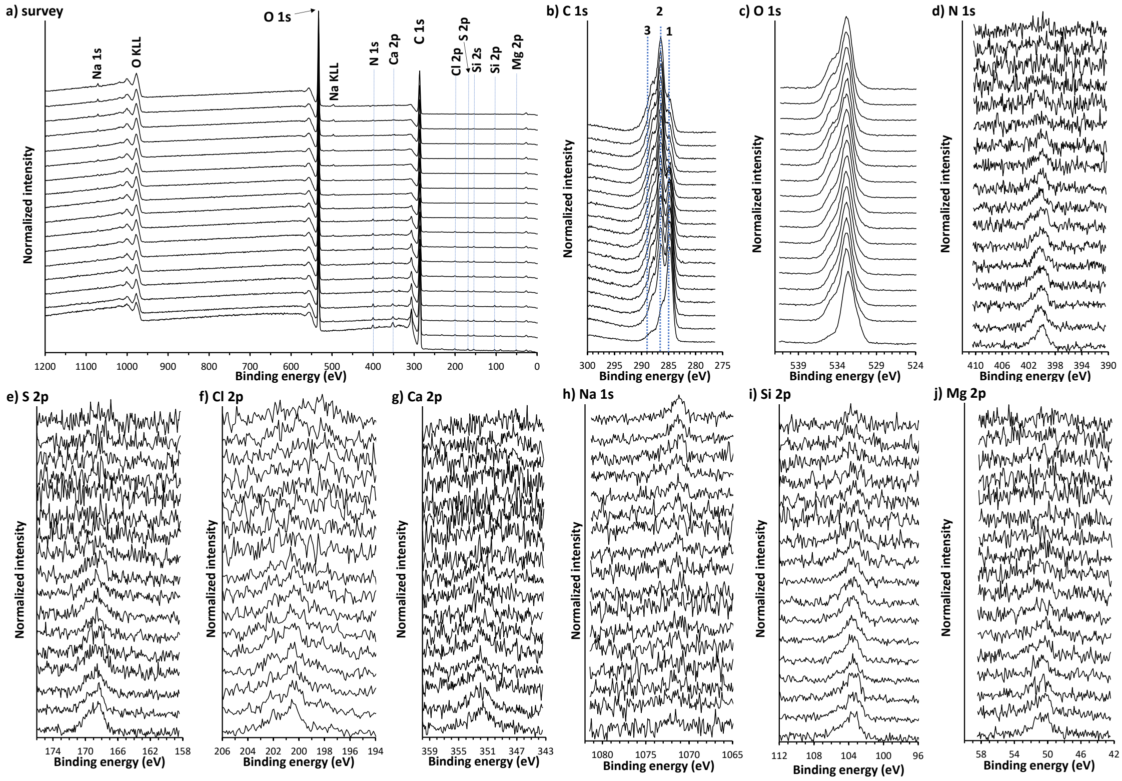

Figure S17: XPS analysis of the COMB 4 tablet: a) survey XPS spectra and HR spectra for b) C 1s, c) O 1s, d) N 1s, e) S 2p, f) Cl 2p, g) Ca 2p, h) Na 1s, i) Si 2p, and j) Mg 2p. Further details are as presented in Figure S14.

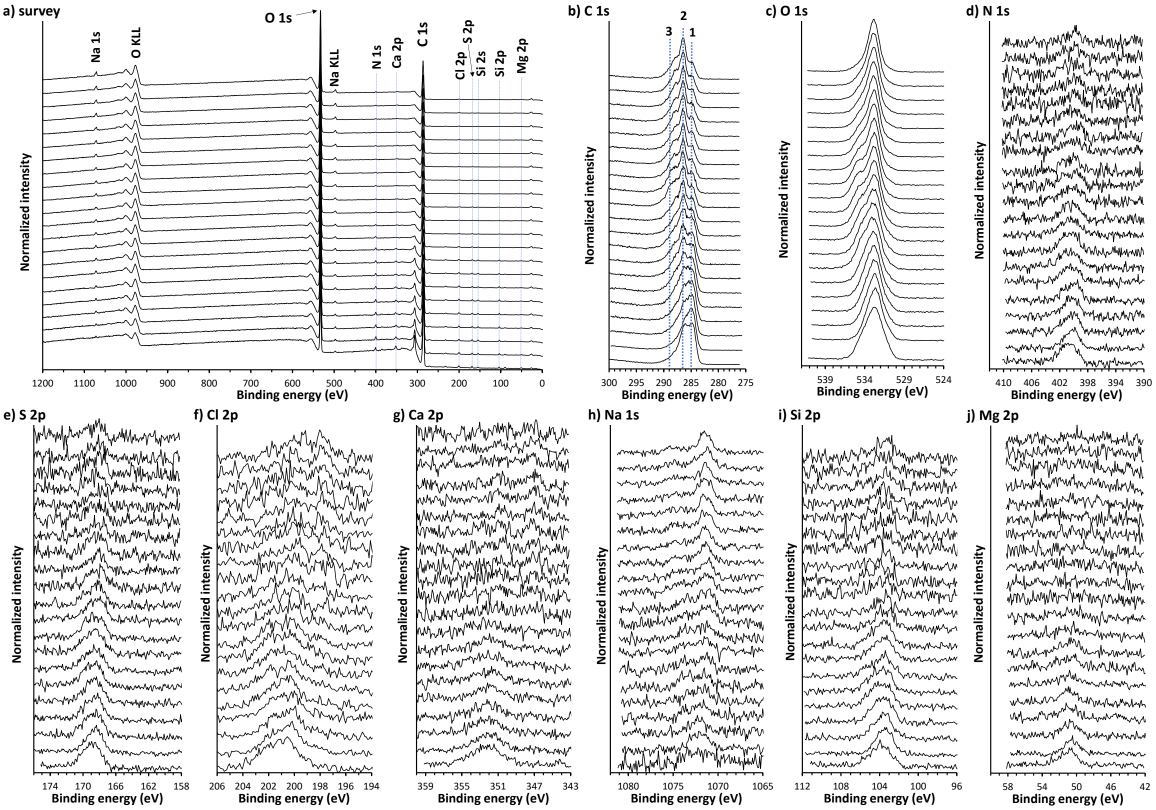

Figure S18: XPS analysis of the COMB 5 tablet: a) survey XPS spectra and HR spectra for b) C 1s, c) O 1s, d) N 1s, e) S 2p, f) Cl 2p, g) Ca 2p, h) Na 1s, i) Si 2p, and j) Mg 2p. Further details are as presented in Figure S14.

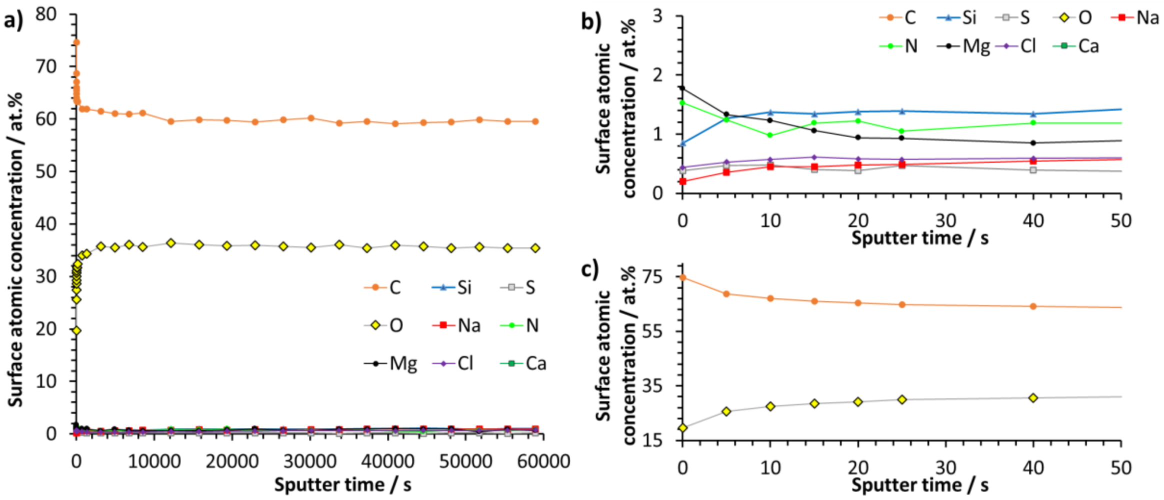

Figure S19: XPS depth profiles of the COMB 1 tablet: a) the extended sputter time, b) the reduced sputter time with a narrower surface atomic concentration scale, and c) the reduced sputter time with a broader surface atomic concentration scale.

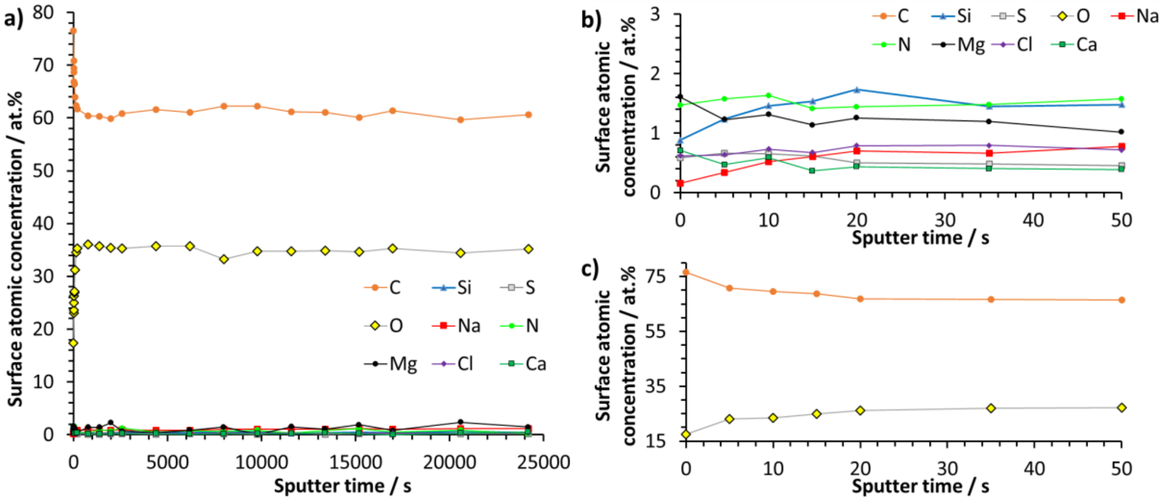

Figure S20: XPS depth profiles of the COMB 2 tablet: a) extended sputter time, b) reduced sputter time with a narrower surface atomic concentration scale, and c) reduced sputter time with a broader surface atomic concentration scale.

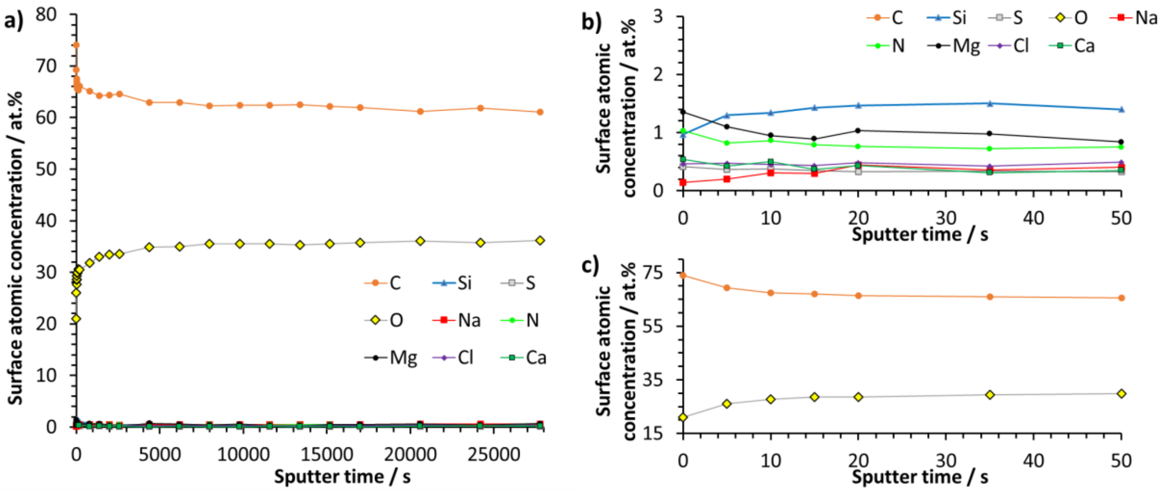

Figure S21: XPS depth profiles of the COMB 3 tablet: a) extended sputter time, b) reduced sputter time with a narrower surface atomic concentration scale, and c) reduced sputter time with a broader surface atomic concentration scale.

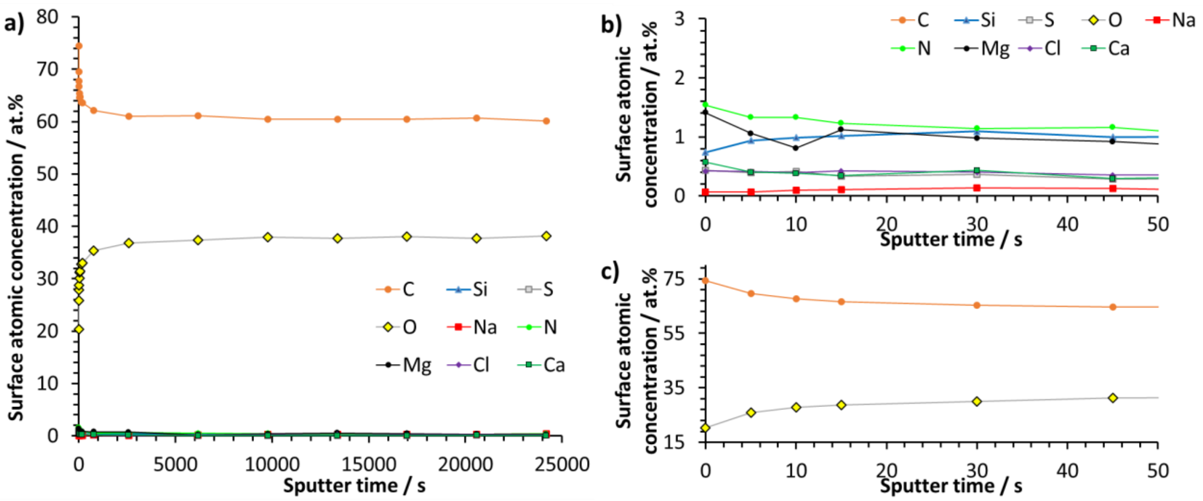

Figure S22: XPS depth profiles of the COMB 4 tablet: a) extended sputter time, b) reduced sputter time with a narrower surface atomic concentration scale, and c) reduced sputter time with a broader surface atomic concentration scale.

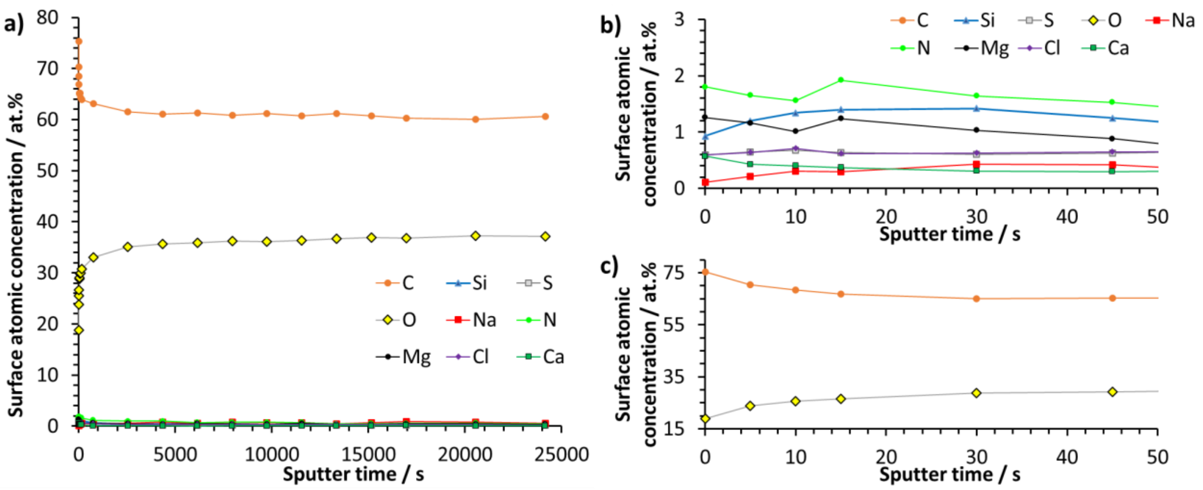

Figure S23: XPS depth profiles of the COMB 5 tablet: a) extended sputter time, b) reduced sputter time with a narrower surface atomic concentration scale, and c) reduced sputter time with a broader surface atomic concentration scale.
